# Neuroanatomical and clinical factors predicting future cognitive impairment

**DOI:** 10.1007/s11357-024-01310-0

**Published:** 2024-08-17

**Authors:** Phoebe Imms, Nikhil N. Chaudhari, Nahian F. Chowdhury, Haoqing Wang, Xiaokun Yu, Anar Amgalan, Andrei Irimia

**Affiliations:** 1 Leonard Davis School of Gerontology, University of Southern California, 3715 McClintock Ave, Los Angeles, CA 90089 USA; 2 Department of Biomedical Engineering, Viterbi School of Engineering, Corwin D. Denney Research Center, University of Southern California, 1042 Downey Way, Los Angeles, CA 90089 USA; 3 Computer Science Department, School of Engineering, Columbia University, Mailing Address: 500 West 120 Street, Room 450, New York, NY MC040110027 USA; 4 Department of Quantitative & Computational Biology, Dana and David Dornsife College of Arts & Sciences, University of Southern California, Mailing Address: 3620 S Vermont Ave, Los Angeles, CA 90089 USA

**Keywords:** Alzheimer’s disease, Machine learning, Early identification, Diagnosis, Prediction, Magnetic resonance imaging

## Abstract

Identifying cognitively normal (CN) older adults who will convert to cognitive impairment (CI) due to Alzheimer’s disease is crucial for early intervention. Clinical and neuroimaging measures were acquired from 301 CN adults who converted to CI within 15 years of baseline, and 294 who did not. Regional volumes and brain age measures were extracted from *T*_1_-weighted magnetic resonance images. Linear discriminant analysis compared non-converters’ characteristics against those of short-, mid-, and long-term converters. Conversion was associated with clinical measures such as hearing impairment and self-reported memory decline. Converters’ brain volumes were smaller than non-converters’ across 48 frontal, temporal, and subcortical structures. Brain age measures of 12 structures were correlated with shorter times to conversion. Conversion prediction accuracy increased from 81.5% to 90.5% as time to conversion decreased. Proximity to CI conversion is foreshadowed by anatomic features of brain aging that enhance the accuracy of predicting conversion.

**Supplementary Information:**

The online version contains supplementary material available at 10.1007/s11357-024-01310-0.

## Background

Up to 40% of dementia cases could be prevented by targeting modifiable risk factors [1]. Treatment during the pre-clinical phase of cognitive impairment (CI) due to Alzheimer’s disease (AD), when neurological changes occur without symptoms [2], could be key to AD prevention and to the improvement of clinical trial efficacy [3]. Thus, early identification of future converters to CI among cognitively normal (CN) older adults is important [4]. Currently, future converters to CI cannot be identified using routine neurocognitive testing or clinical evaluation. However, neuroimaging holds potential to yield early insight on AD risk because AD-related structural brain changes precede conversion by up to 20 years [3, 5]. *T*_1_-weighted magnetic resonance imaging (MRI) allows the quantitation of structural brain morphometry, whose alterations may reflect AD neuropathology [6]. Questions remain about the temporality of such changes [7], as well as about the reversibility and preventability of neurodegeneration before CI conversion becomes inevitable. This study compares the clinical and neurological profiles of (A) CN adults who convert to MCI or AD within 15 years of baseline assessment (converters), with those of (B) CNs who do not convert within this period (non-converters). We also quantify the ability to predict future CI conversion as a function of remaining time before conversion. Hereinafter, ‘CI conversion’ is a new diagnosis of either mild CI (MCI) or AD within 15 years, rather than beyond this time frame, and ‘CI status’ is an older adult’s current diagnosis (either CN or CI).

Approximately 72% of future CI converters can be identified using machine learning (ML) of known clinical, health, and demographic risk-factors for AD [8]. This sensitivity may be enhanced by the inclusion of neuroimaging measures that reflect CI-related atrophy and brain aging. Neuropathological changes that occur before CI conversion frequently involve the hippocampus, amygdala, entorhinal cortex, and medial temporal lobe [3]. Altered brain morphometry in CN older adults has been observed ~ 5 years before they convert to MCI [9], and 7–10 years before AD [10, 11], mirroring the common diagnostic trajectory of CN to MCI to AD. CI-related patterns of atrophy correlate strongly with neuropsychological deficits [12, 13] and map accurately onto Braak stages of neurofibrillary tangle deposition [14–17]. Studies predicting CI status from *T*_1_-weighted MRI have focused predominantly on subjects who convert from MCI to AD [e.g., 18, 19, 20, for review, see 21], and on cohorts of fewer than 150 patients [e.g., 22, 23, 24]. Differences in brain structure between CN older adults with and without CI conversion in their future are far less evident in the literature, but substantially more useful for early targeted intervention.

Aside from volumetrics, we use ML of *T*_1_-weighted MRIs to estimate brain age (BA; the biological age of the brain) and BA saliency (the importance of brain regions to ML age estimation). We do so because average BA is significantly older in MCI or AD [25], useful to predict conversion from MCI to AD [26], and sensitive to cognitive decline [27]. Converters are hypothesized to have more conspicuous aging-related brain features (e.g., smaller regional brain volumes, higher BA saliencies) than non-converters, reflecting brain aging differences that predicate CI conversion. To relate neuroanatomic features of aging to converters’ proximity of conversion, we correlate these features with time to conversion. We also use these features to distinguish non-converters from older adults who convert in the short-term (ST), mid-term (MT), and long-term (LT). This work contributes to ongoing efforts to determine whether conversion is preventable, suggesting that neurodegenerative changes accelerate ahead of CI conversion, becoming more apparent and less reversible as conversion becomes more proximate. This study suggests that, on average, older adults less than 2.5 years away from conversion have neared the critical point of no return along their structural brain trajectories toward AD. It may be around this critical point that brain changes indicative of conversion become sufficiently easy to detect from MRI.

## Methods

### Participants

Procedures performed were in accordance with the ethical standards of the 1964 Helsinki Declaration and its later amendments, the US Code of Federal Regulations (45 CFR 46), and with approval from the Institutional Review Board of the University of Southern California. Written informed consent was obtained from all participants (or guardians of participants). Data were obtained from the National Alzheimer's Coordinating Center (NACC), which is responsible for maintaining patient information from 37 AD research centers (ADRCs) funded by the National Institute on Aging (NIA) [28, 29]. This study used data from 20 ADRCs’ standardized Uniform Datasets (UDSs) collected between September 2005 and March 2023. Details have been published on NACC’s neuropsychological [30, 31] and clinical assessments [32–34]. Qualified researchers may apply for access to all neuroimaging and clinical data used in this study (https://naccdata.org/).

Data were from 3,020 adults who had at least one *T*_1_-weighted MRI with associated neuropsychological visit (baseline), at least one further neuropsychological visit (follow-up), and who were CN at baseline. CN status was determined by consensus-based UDS diagnosis (UDSD) of normal cognition (NACC UDSD = 1, ‘normal cognition’). CI status (at follow-up) was determined by consensus-based diagnosis of MCI (NACC UDSD = 3) or dementia (NACC UDSD = 4) *with* presumptive etiology of AD (NACC ALZD = 1). Consensus-based diagnoses utilized AD presumptive etiology according to the National Institute of Neurological and Communicative Disorders and Stroke, the Alzheimer’s Disease and Related Disorders Association and the NIA & Alzheimer’s Association criteria. Subjects with ‘other’ causes of CI (e.g., Parkinson’s disease, Lewy body dementia, vascular dementia, mixed etiologies) were excluded (*N* = 312). At follow-up, 442 subjects were diagnosed with CI due to AD (either MCI with AD as presumptive etiology, or AD); 408 transitioned from CN to MCI, and 34 from CN to AD. Also excluded were 711 subjects who did not have clinical data, leaving 1,696 non-converters (i.e., CNs at follow-up within the 15-year time frame), and 301 converters. A subject was considered a converter as soon as they had a CI (either MCI or AD) diagnosis, even if they reverted to CN (35, or 11.62%, reverted to CN by the end of their time in the study). Time in the study was counted as the number of years from baseline to follow-up. For converters, follow-up was the visit where conversion to MCI or AD had been first recorded; for non-converters, it was their last visit on record.

#### Subject matching

Conversion is biased by age, as older adults are more likely to be diagnosed with CI. Thus, converters are, on average, older than non-converters. Case–control matching was used to pair each converter to a non-converter according to age (within 2 years), sex, and time in the study (within 2 years). For example, a CN female aged 75 at baseline, diagnosed with MCI at 78, was matched with a female aged 75 ± 2 years at baseline who, at follow-up, remained CN at age 78 ± 2 years. The matching ratio was set at 1:1 to balance the sample. The final sample included 595 neurologically healthy adults (301 converters and 294 non-converters, 372 females), aged between 47 and 100 years at baseline, and between 52 and 105 years at follow-up. Seven converters could not be matched to non-converting CN participants due to the CN sample size.

### Clinical variables

The NACC database contains over 750 clinical measures relating to subjects’ demographics, family history, health history, physical health, and neurocognitive status (interviews, questionnaires, cognitive batteries, and neurological symptoms). We selected 42 of these based on (A) feature extraction from random forest/logistic regression classifiers predicting conversion from CN to CI using NACC clinical variables [8], and (B) review articles expounding important CI risk factors in CNs [1, 35]. We excluded clinical variables with more than 5% missing data. Supplementary Table A lists all variables included, how each was measured, its data type and measurement scale, and the literature sources supporting its inclusion. Each variable was categorized as *demographic* (e.g., age at baseline, sex, years of education), *health* (e.g., body mass index, smoking status, history of heart attack or stroke, hearing impairment, and ApoE-ε4 carrier status), *interview-based measure of neurocognitive functioning* (e.g., clinical dementia rating (CDR® Dementia Staging Instrument), self-reported memory impairment, clinician-rated cognitive status), or as a *neurocognitive test score* (e.g., digit span, verbal fluency, trails A & B).

Neurocognitive tests that quantify the same cognitive function but changed between UDS v1.2/v2.0 and UDS v3.0/v3.2 were combined, allowing the inclusion of subjects across all UDSs since their inception in 2005. For example, logical memory scores (v1.2/v2.0) and Craft story 21 scores (v3.0/v3.2) were combined (hereinafter referred to as ‘story recall’). The Boston naming (v1.2/v2.0) and multilingual naming tests (v3.0/v3.2) were combined (hereinafter referred to as ‘picture naming’). The digit span (v1.2/v2.0) and number span tests (v3.0/v3.2) were also combined (hereinafter referred to as ‘digit span’). Scores were adjusted across combined tasks by converting each to the percentage of correct answers out of the total possible number of correct answers (see Supplementary Table A, ‘scale’ for details).

### Brain volumes and age saliencies

#### ***T***_1_***-weighted image acquisition and processing***

MRIs were voluntarily submitted to NACC between 2005 and March 2023. *T*_1_-weighted image acquisition protocols vary across the 20 ADRCs included in this study. DICOMs were de-identified prior to distribution by NACC. Quality control by visual assessment was performed before and after Freesurfer (https://surfer.nmr.mgh.harvard.edu/, version 6.0.0) processing, which included removal of non-brain tissues, transformation into Talairach space, intensity normalization, segmentation into cortical/subcortical structures, surface processing, and topology correction. Structure-level volumetrics were computed for 185 brain structures (148 cortical, 19 subcortical, 9 white matter, and 9 cerebrospinal fluid (CSF); see Supplementary Table B) according to the Destrieux brain atlas [36].

#### Allometric scaling

Brain structures scale non-proportionally to total intracranial volume [TICV; 37]. For this reason, instead of linearly normalizing brain volumes to TICV, volumes were allometrically scaled. The natural logarithm of each brain structure was regressed on age, sex, and the natural logarithm of the TICV to produce the allometric scaling coefficient *β*. This coefficient was used in the following equation from Kaplan et al. [38] to compute the normalized percentage of brain volume (hereinafter referred to as brain volume, BV), for each brain structure, in subject *i*:

$$B{V}_{i} [\%]= {BV}_{i}\times \frac{{[{\mu }_{i}(TIC{V}_{i})]}^{\beta -1}}{{TICV}_{i}^{\beta }}$$

Above, the mean $\mu$ is computed over all $i$. After allometric correction, the total volumes of cortical grey matter, subcortical grey matter, white matter, and CSF were the respective sums of these structures’ volumes. For example, total cortical grey matter was the sum of all 148 cortical structures’ volumes.

#### Brain age and saliency

BA was estimated using a deep neural network (DNN), which is among the most accurate, interpretable, and generalizable methods available [25]. Its architecture, training, and performance are described elsewhere [25]. Briefly, a DNN was used to estimate BA by learning CN subjects’ CAs from MRIs of their brain, while minimizing the mean squared error difference between BA and CA. DNN inputs are the subjects’ CAs and their *brain.mgz* files from Freesurfer containing skull-stripped *T*_1_-weighted MRIs. Outputs are BA, age gap (AG, where ${AG}_{i}={BA}_{i}-{CA}_{i}$ for each subject *i*), and BA *saliency maps*. Saliency maps are organized topographically according to how salient (i.e., important) anatomic locations are to the DNN when estimating BA. Such maps reveal neuroanatomic patterns of aging. Brain structures that are more useful during biological age estimation have higher saliency, and those less important have lower saliency. One can consider brain structures with higher saliency to be more entrained in the aging process, in the sense that such structures highlight key features of brain aging [25]. Saliency at each brain location (voxel) was divided by the sum of all brain saliencies, thereby operationalizing saliency as a saliency probability. Cortical saliency probability maps were projected onto a cortical/subcortical overlay using custom volume-to-surface mapping [39, 40]. We computed average BA saliency probability (hereinafter referred to simply as saliency) for each Freesurfer atlas brain structure.

### Statistical analysis

#### Demographics, health measures, and neurocognitive assessments

The average clinical presentation of converters were compared to that of non-converters using *t*-tests and χ^2^ goodness-of-fit tests. Clinical variables that were continuous (rather than categorical) were compared between converters and non-converters using Student’s *t*-tests and Cohen’s *d* (small effect: *d* < 0.2; medium effect: 0.2 < *d* < 0.5; large effect: *d* > 0.5) [41] to estimate effect sizes. Where equal variances could not be assumed, Welch’s *t*-tests and Glass’s *Δ* were used instead. For categorical variables, χ^2^ goodness-of-fit tests were used to judge the significance of discrepancies between expected and observed counts for converters vs. non-converters. All results were corrected for multiple comparisons using the false discovery rate (FDR) method [42].

#### Regional brain volumes and saliencies

Converters and non-converters’ average whole brain volumes and saliencies for cortical grey matter, subcortical grey matter, white matter, and CSF were compared using independent-sample* t*-tests. The association between (A) time to conversion and (B) whole brain volumes or saliencies was examined using Pearson’s partial correlation *r*, controlling for age at baseline, sex, and years of education. To examine *regional* differences in volume and saliency between converters and non-converters, and their correlations with time to conversion, the same *t*-tests and partial correlations were performed separately for each of the 185 segmented structures’ volumes and saliencies. All results were FDR corrected.

### Classification of converters and non-converters

We attempted to distinguish converters from non-converters using linear discriminant analyses (LDAs) implemented in IBM SPSS v28.0.0. To reduce the number of anatomic features and minimize overfitting, brain regions’ features were included only if they were (A) significantly different between converters and non-converters, or (B) correlated with time to conversion. Five analyses were executed using different feature combinations: (A) clinical variables alone (43 features), (B) brain volumes alone (48 features), (C) saliencies alone (12 features), (D) clinical variables and brain volumes (91 features), (E) clinical variables and saliencies (55 features), and (F) clinical variables, brain volumes, and saliencies (103 features). Overfitting was assessed using leave-one-out cross-validation. Wilks’ $\lambda$ (i.e., the proportion of total variance in the discriminant scores not explained by differences between groups) and its associated χ^2^ test statistic were computed. These were used as indicators of whether the discriminant function did better than chance at separating converters from non-converters. Features that were most integral for classification were also identified in the structure matrix, which specifies the correlation of each feature with the discriminant function. We extracted sensitivity, specificity, precision, and accuracy from LDA classification tables.

#### Classifying ST, MT, and LT converters

We investigated whether ST converters could be classified more accurately than MT or LT converters. Terciles of times to conversion were calculated from their cumulative distribution function to determine cut-offs for ST (conversion within 2.42 years of assessment), MT (conversion between 2.43 and 5.17 years), and LT (conversion between 5.18 and 15 years) converters. LDAs distinguished converters according to type (ST, MT, or LT) from a matched sample of 92 non-converters. For baseline comparison with the same sample size, LDAs distinguished non-converters from three random samples of converters who were *not* stratified by time to conversion. Classification measures (i.e., sensitivity, specificity, precision, and accuracy) were averaged across these three analyses.

## Results

### Demographic, health, and neurocognitive measures across converters and non-converters

**Fig. 1:**
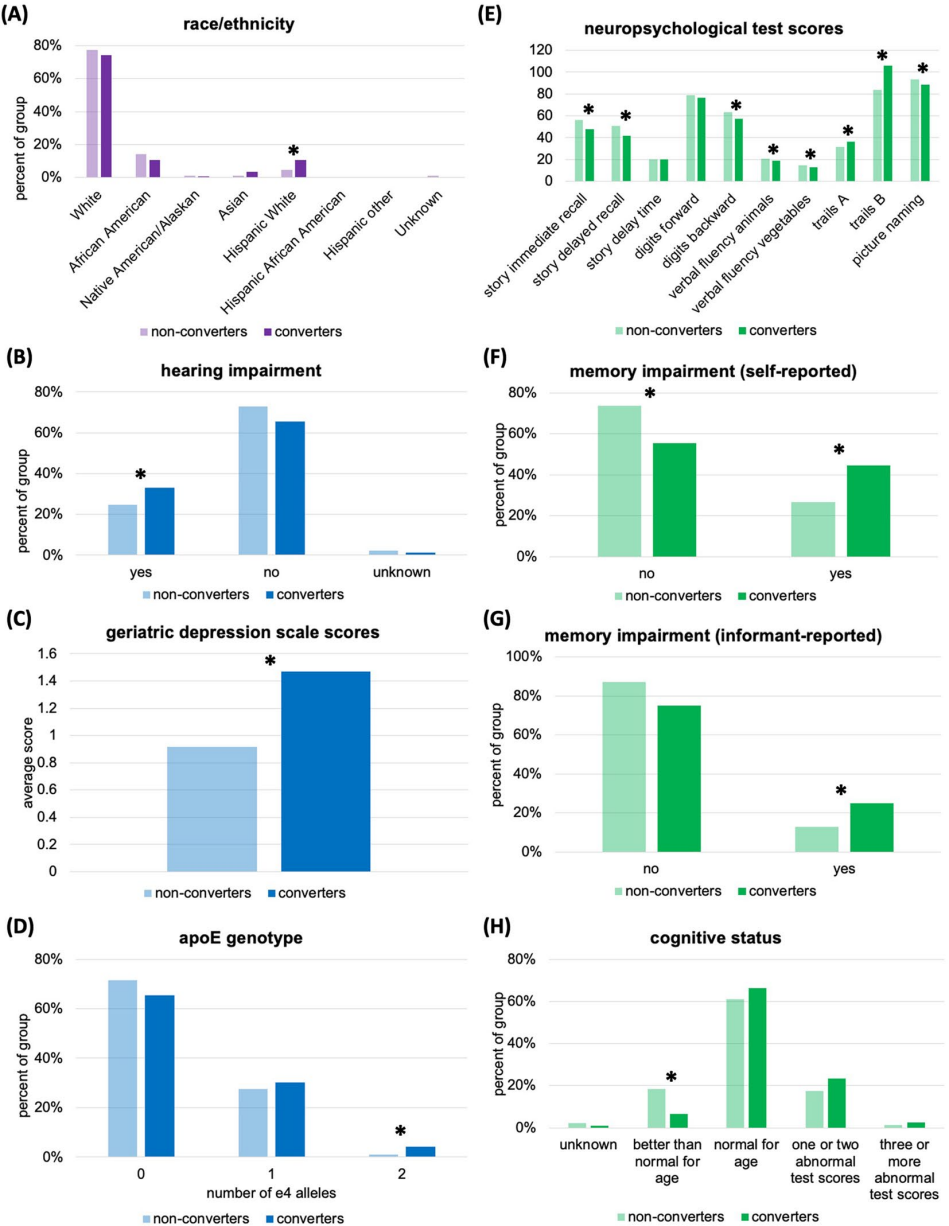
Comparison of clinical AD risk factors between converters (dark colors) and non-converters (light colors). (**A**) Race/ethnicity representations. *Note: Races/ethnicities with no representation in either group are not displayed, including Hawaiian and Pacific Islanders.* (**B**) Percentage of subjects whose hearing is impaired (‘yes’) or functions normally (‘no’) without a hearing aid. (**C**) Average geriatric depression scale scores. (**D**) Percentage of subjects with zero, one, or two *ε4* alleles. (**E**) Average neuropsychological test scores. Scores for *story immediate recall, story delayed recall, digits forward, digits backward,* and *picture naming* are expressed as percentages of correct responses out of the total possible number of correct responses. Time is the score value for *story delay time* (minutes)*, trails A* (seconds)*, and trails B* (seconds). *Verbal fluency animals* and *verbal fluency vegetables* scores are total counts of correct responses. (**F**) Percentage of subjects with self-reported memory impairment. (**G**) Percentage of subjects with memory impairment as reported by an informant. (**H**) Cognitive status of subjects based on a summary of neuropsychological test scores. Asterisks indicate that differences between converters and non-converters were significant, as assessed using Students’ *t*-test for independent samples (*p* < 0.05, FDR corrected, for (C) or (E)) or χ2 test of association for (A), (B), (D), (F), (G), and (H)

Since subjects had been matched by age at baseline and sex, no significant differences between converters and non-converters were observed in these measures ($p$ > 0.05, corrected). Time in the study trended towards significance; the time between baseline and follow-up for non-converters (*μ* = 4.52 years) was slightly longer than for converters (*μ* = 4.42 years, *t*_(595)_ = 1.45, *p* = 0.15). Converters were diagnosed with CI (either MCI or AD) at an average age of 80.49 ± 8.75 (μ ± σ) years (Table 1). Converters had significantly higher geriatric depression scale (GDS) scores (*μ* = 1.49, σ = 3.14) than non-converters (*μ* = 0.92, *σ* = 1.67, *t*_(595)_ = -3.68, *p* < 0.001; Fig. 1 (C)), indicating higher levels of self-reported depressive symptoms in the former. There was a trend for converters’ BAs (*μ* = 74.89) to be slightly older than non-converters’ (*μ* = 73.93, *t*_(595)_ = -1.36, *p* = 0.18). On average, both converters and non-converters’ mean AGs were negative, i.e., their BAs were younger than their CAs. There was a trend for non-converters’ AGs (*μ* = -1.51) to be slightly further from zero than converters’ (*μ* = -1.19, *t*_(595)_ = -1.60, *p* = 0.11), indicating that the former’s brain aging was slower than the latter’s. There was no significant difference between converters and non-converters in BA, years of education, or body mass index.

**Table 1:** Comparison of demographics and (continuous) health variables across converters and non-converters^†^

	**non-converters (*N*** **= 294)**			**converters (*N*** **= 301)**			**non-converters vs. converters**	
	*μ (σ)*	*min*	*max*	*μ (σ)*	*min*	*max*	*t*_*(595)*_	*p*
**baseline age**	75.40 (8.02)	48.33	95.83	76.07 (8.34)	47.92	100.25	–1.00	0.32
**age at CI dx**				80.49 (8.75)	49.75	104.58		
**brain age**	73.93 (8.37)	46.75	98.05	74.89 (8.84)	43.74	104.78	–1.36	0.18
**age gap**	–1.51 (2.32)	–14.63	7.90	–1.19 (2.61)	–17.76	8.15	–1.60	0.11
**education**	16.04 (2.79)	6.00	25.00	15.69 (3.14)	1.00	22.00	0.41	0.68
**time in study**	4.52 (2.93)	0.67	13.83	4.42 (3.14)	0.50	14.75	1.45	0.15
**GDS**	0.92 (1.67)	0.00	14.00	1.49 (2.08)	0.00	13.00	–3.68	< 0.01*
**BMI**	27.09 (5.18)	16.60	46.90	27.29 (5.11)	18.10	52.40	–0.47	0.32

*Significant after FDR correction. Values in **bold** are significant at *p* < 0.05

^†^All values are in years except for GDS and BMI, which are dimensionless

*CI* cognitive impairment; *dx* diagnosis; *GDS* geriatric depression scale, *BMI* body mass index

#### Demographic and health-related factors for conversion

Supplementary Table C lists χ^2^ comparisons between converters and non-converters for categorical clinical variables (demographic, health, and interview-based neuropsychological measures). Future conversion was significantly associated with race/ethnicity (*χ*^2^_(7)_ = 16.30, *p* = 0.02): converters were more likely to be Hispanic White (10.63%) than non-converters (4.76%, *χ*^2^_(1)_ = 6.58, *p* = 0.01; Fig. 1 (A)). There were no significant associations between future conversion and handedness, marital status at baseline, or independence of living at baseline. Future conversion was significantly associated with ApoE genotype (*χ*^2^_(2)_ = 7.165, *p* = 0.03): more converters (4.32%) compared to non-converters (1.02%) had two *ε4* alleles than expected by chance (*χ*^2^_(1)_ = 6.00, *p* = 0.01, Fig. 1 (D)). Future conversion was also significantly associated with hearing loss (*χ*^2^_(1)_ = 4.74, *p* = 0.03): more converters (33.22%) than non-converters (24.83%) had hearing that was not functionally normal without hearing aid(s) (*χ*^2^_(1)_ = 3.89, *p* = 0.049; Fig. 1 (B)). There were no significant associations between future conversion and current smoking habits, alcohol abuse, history of cardiac arrest, history of stroke, history of hypertension, history of traumatic brain injury [43], or diabetes.

#### Converters have early neuropsychological indicators of CI

Converters were less likely than non-converters to have neurocognitive test scores ‘better than normal for their age’, as determined by a clinician. A total of 18.4% of non-converters met this criterion, but only 6.6% of converters had better-than-normal cognitive test scores (*χ*^2^_(1)_ = 16.37, *p* =  < 0.001; Fig. 1 (H); Supplementary Table C). One converter had a global CDR score of 1 indicating mild impairment, however they had no abnormal neuropsychological test scores. Thus, their cognitive status was deemed ‘normal for their age’ by the clinician. All other subjects had global CDR scores of 0.5 (“questionable impairment”, 18.3% of converters and 14.6% of non-converters) or 0 (“no impairment”, 81.4% of converters and 85.4% of non-converters). There was no significant association between future conversion and global CDR score. While there was no significant association between future conversion and clinician assessed memory impairment, there was a strong association between future conversion and *self-reported* memory impairment (*χ*^2^_(1)_ = 21.329, *p* < 0.001; Fig. 1 (F), Supplementary Table C), and with *informant-reported* memory impairment (*χ*^2^_(1)_ = 14.111, *p* < 0.001; Fig. 1 (G), Supplementary Table C). Almost half (44.1%) of converters reported that their memory was meaningfully impaired relative to their previous abilities, while only 26.5% of non-converters said the same (*χ*^2^_(1)_ = 13.721, *p* =  < 0.001). One quarter of converters’ informants (25.1%) endorsed memory impairments, while only 12.8% of non-converters’ informants did (*χ*^2^_(1)_ = 11.370, *p* =  < 0.001). There were no significant associations between future conversion and subjects’ judgement, community engagement, visuospatial function, or symptoms of cognitive, behavioural, and motor impairment.

At baseline, converters had worse neuropsychological test scores than non-converters (Supplementary Table D), in the cognitive domains of logical memory recall (immediate and delayed), digit span backwards, verbal fluency, executive function, and confrontation naming (language ability) (Fig. 1 (E)). There was no significant difference between converters’ and non-converters’ digit span forwards or in their delay time between immediate and delayed story recall. Despite converters performing worse *on average*, only 8 converters (and 4 non-converters) had more than 2 abnormal test scores (i.e., scores below the cut-off for their age, sex, and educational status) (*χ*^2^_(1)_ = 1.20, *p* = 0.27).

### Future conversion, brain volume, and saliency

Converters had significantly smaller total cortical grey matter volumes (*t* = 3.625, *p* < 0.001) and significantly larger total CSF volumes (*t* = -2.690, *p* = 0.007) than non-converters (Table 2). Total brain volumes were not significantly associated with time to conversion after FDR correction. In converters, higher saliency of the total cortical grey matter was associated with shorter times to conversion (Table 2, *r* = -0.17, *p* = 0.003). No significant differences in saliency of the total cortical grey matter, total white matter, or total CSF were observed between converters and non-converters after FDR correction.

**Table 2:** Comparison of total grey matter, white matter, and cerebrospinal fluid volumes and saliencies for converters and non-converters, including partial correlations with time to conversion

	**non-converters** *μ (σ)*	**converters** *μ (σ)*	***t***	***df***	***p***	***r***	***p***
**volume**^†^							
*CSF*	2.620 (1.044)	2.869 (1.216)	–2.690	583.347	0.007*	–0.097	0.096
*WM*	30.190 (3.267)	30.894 (5.037)	–2.026	516.042	**0.043**	0.125	**0.032**
*subcortical GM*	11.657 (0.780)	11.455 (1.304)	2.227	526.902	**0.026**	0.054	0.349
*cortical GM*	28.932 (2.299)	28.100 (3.234)	3.625	542.173	<0.001*	–0.006	0.916
**saliency**							
*CSF*	0.017 (0.005)	0.016 (0.005)	1.648	592.999	0.100	–0.032	0.579
*WM*	0.008 (0.002)	0.006 (0.002)	1.028	590.007	0.304	–0.067	0.250
*subcortical GM*	0.022 (0.005)	0.021 (0.005)	2.307	592.955	**0.021**	–0.120	**0.038**
*cortical GM*	0.100 (0.022)	0.098 (0.024)	1.280	591.093	0.201	–0.170	0.003*

*Significant after FDR correction. Values in **bold** are significant at *p* < 0.05

^†^Volume is expressed in percent of the total intracranial volume (TICV, mm^3^), allometrically corrected for age and sex

*GM* grey matter, *WM* white matter, *CSF* cerebrospinal fluid

#### Converters’ regional brain volumes are smaller than non-converters’

**Fig. 2:**
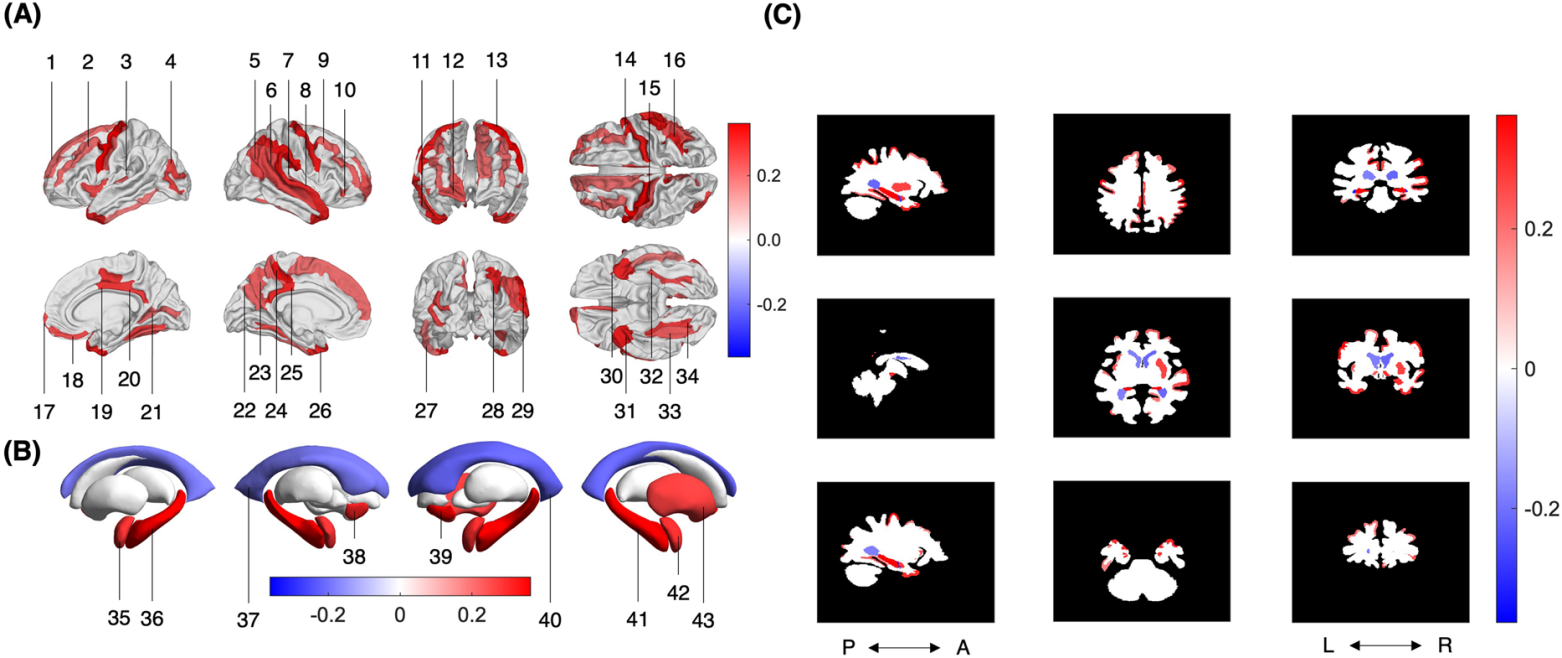
Structures whose volumes differ significantly between converters and non-converters, after FDR correction. (**A**) Cortical, (**B**) subcortical, (**B**) & (**C**) cerebrospinal fluid, and (C) white matter. Neurological convention is used in (C). Converters had smaller grey matter volumes than non-converters, and larger CSF volumes. 1 = left superior frontal gyrus; 2 = left middle frontal gyrus; 3 = left anterior transverse temporal gyrus; 4 = left middle occipital gyrus; 5 = right sulcus intermediate primus; 6 = right temporal plane of the superior temporal gyrus; 7 = right anterior transverse temporal gyrus; 8 = right lateral aspect of the superior temporal gyrus; 9 = right middle frontal gyrus; 10 = right orbital part of the inferior frontal gyrus; 11 = right superior temporal sulcus; 12 = right frontomarginal gyrus and sulcus; 13 = left precentral gyrus; 14 = right precentral gyrus; 15 = left central sulcus; 16 = right intraparietal sulcus and transverse parietal sulci; 17 = right transverse frontopolar gyri and sulci; 18 = right straight gyrus; 19 = right middle posterior part of the cingulate gyrus and sulcus; 20 = right posterior-dorsal part of the cingulate gyrus; 21 = right calcarine sulcus; 22 = left parieto-occipital sulcus; 23 = left precuneus; 24 = left marginal branch of the cingulate sulcus; 25 = left posterior-dorsal part of the cingulate gyrus; 26 = left temporal pole; 27 = left inferior temporal gyrus; 28 = right angular gyrus; 29 = right supramarginal gyrus; 30 = left short insular gyri; 31 = right temporal pole; 32 = left medial occipito-temporal sulcus and lingual sulcus; 33 = right lateral occipito-temporal gyrus; 34 = right medial occipito-temporal sulcus and lingual sulcus; 35 = left amygdala; 36 = left hippocampus; 37 = right lateral ventricle; 38 = right accumbens; 39 = left accumbens; 40 = left lateral ventricle; 41 = right hippocampus; 42 = right amygdala; 43 = right putamen. *Not pictured: left inferior lateral ventricle, right inferior lateral ventricle, left ventral diencephalon, right ventral diencephalon*

After FDR correction, converters had 34 smaller cortical structures than non-converters, including 10 frontal, 8 temporal, 7 parietal, 5 occipital, and 4 limbic (Table 3 and Fig. 2(A)). Nine subcortical structures were also smaller in converters compared to non-converters, including both nuclei accumbentes, amygdalae, hippocampi, ventral diencephalons, as well as the right putamen (Fig. 2(B)). Both lateral ventricles were significantly larger in converters compared to non-converters Fig. 2(C). No significant differences in white matter volume were observed between converters and non-converters. After averaging the sum of all cortical gray matter volumes across participants, 13.0% of converters’ TICVs were found to be significantly smaller than non-converters’. The largest differences observed were in the following structures (ranked from largest to smallest Cohen’s *d*, where *d* > 0.3 indicates a moderate effect size): right hippocampus (*d* = 0.41), left hippocampus (*d* = 0.36), right accumbens (*d* = 0.36), left precentral gyrus (*d* = 0.35), right supramarginal gyrus (*d* = 0.33), left marginal branch of the cingulate sulcus (*d* = 0.33), right precentral gyrus (*d* = 0.32), right temporal plane of the superior temporal gyrus (*d* = 0.32), right temporal pole (*d* = 0.31), right intraparietal sulcus and transverse parietal sulci (*d* = 0.31), left accumbens (*d* = 0.31), and left temporal pole (*d* = 0.30). No significant differences in brain structure saliency were observed between converters and non-converters (Table 4).

**Table 3:** Differences between converters’ and non-converters’ regional volumes and their correlations with time to conversion. Only structures that survived FDR correction are listed

**lobe**	**structure**	**non-converters** ***μ(σ)***	**converters** ***μ(σ)***	***d*** ^***†***^	***t***	***df***	***p***	**1−*β***	***r***	***p***
**lateral ventricles**	*L inferior*	0.04 (0.03)	0.05 (0.03)	**–0.33**	–3.46	575.46	0.001*	0.97	–0.13	**0.029**
	*R inferior*	0.05 (0.03)	0.05 (0.03)	–0.29	–3.02	573.38	0.003*	0.92	–0.11	**0.048**
	*R*	1.04 (0.46)	1.14 (0.53)	–0.21	–2.54	586.33	0.012*	0.77	–0.08	0.186
	*L*	1.17 (0.52)	1.29 (0.61)	–0.18	–2.51	581.74	0.013*	0.78	–0.11	0.070
**frontal**	*L precentral G*	0.37 (0.05)	0.35 (0.06)	**0.35**	4.19	585.45	< 0.001*	0.99	–0.10	0.082
	*R precentral G*	0.37 (0.05)	0.36 (0.06)	**0.32**	3.11	585.04	0.002*	0.92	–0.08	0.192
	*R orbital part of the inferior frontal G*	0.06 (0.01)	0.06 (0.01)	0.24	2.67	593.00	0.008*	0.77	0.01	0.929
	*R straight G*	0.14 (0.02)	0.13 (0.03)	0.24	2.63	563.28	0.009*	0.86	–0.02	0.731
	*R frontomarginal G and S*	0.11 (0.02)	0.11 (0.02)	0.23	2.72	571.53	0.007*	0.87	–0.02	0.675
	*R transverse frontopolar gyri and sulci*	0.17 (0.02)	0.16 (0.03)	0.23	2.99	569.55	0.003*	0.92	–0.09	0.115
	*L central S*	0.24 (0.03)	0.24 (0.04)	0.22	2.50	550.73	0.013*	0.85	–0.08	0.181
	*L middle frontal G*	0.61 (0.09)	0.58 (0.10)	0.20	2.85	589.98	0.005*	0.85	0.02	0.772
	*R middle frontal G*	0.55 (0.09)	0.53 (0.09)	0.20	3.17	591.56	0.002*	0.91	0.04	0.445
	*L superior frontal G*	1.05 (0.12)	1.02 (0.14)	0.19	2.67	581.67	0.008*	0.83	–0.03	0.556
**limbic**	*L posterior-dorsal part of the cingulate G*	0.09 (0.02)	0.09 (0.02)	**0.30**	3.99	585.90	< 0.001*	0.99	–0.01	0.819
	*R middle posterior part of the cingulate G and S*	0.16 (0.02)	0.15 (0.03)	0.27	3.79	571.31	< 0.001*	0.99	–0.01	0.826
	*R posterior-dorsal part of the cingulate G*	0.08 (0.01)	0.08 (0.02)	0.26	3.18	562.96	0.002*	0.96	–0.01	0.924
	*L short insular gyri*	0.15 (0.02)	0.14 (0.03)	0.24	3.27	521.74	0.001*	0.99	–0.04	0.535
**occipital**	*R medial occipito-temporal S and lingual S*	0.17 (0.03)	0.17 (0.03)	0.27	3.15	582.41	0.002*	0.93	0.04	0.498
	*L middle occipital G*	0.30 (0.05)	0.28 (0.06)	0.25	3.41	586.20	0.001*	0.95	0.06	0.270
	*L medial occipito-temporal S and lingual S*	0.19 (0.03)	0.18 (0.03)	0.24	3.00	590.35	0.003*	0.88	0.02	0.748
	*R lateral occipito-temporal G*	0.30 (0.05)	0.29 (0.07)	0.22	3.11	564.88	0.002*	0.95	0.06	0.344
	*R calcarine S*	0.18 (0.04)	0.17 (0.04)	0.19	2.73	592.19	0.006*	0.77	0.04	0.505
**parietal**	*L marginal branch of the cingulate S*	0.09 (0.01)	0.09 (0.02)	**0.33**	3.55	590.74	< 0.001*	0.96	–0.01	0.895
	*R supramarginal G*	0.35 (0.05)	0.33 (0.06)	**0.33**	3.16	575.24	0.002*	0.94	–0.03	0.612
	*R intraparietal S and transverse parietal sulci*	0.28 (0.05)	0.27 (0.05)	**0.31**	2.98	583.82	0.003*	0.9	–0.05	0.399
	*R angular G*	0.40 (0.07)	0.39 (0.07)	0.24	3.19	588.34	0.002*	0.92	0.01	0.913
	*R S intermediate primus*	0.04 (0.01)	0.03 (0.01)	0.23	3.29	592.68	0.001*	0.91	–0.01	0.827
	*L precuneus*	0.35 (0.05)	0.34 (0.06)	0.21	2.56	582.71	0.011*	0.79	–0.01	0.857
	*L parieto-occipital S*	0.18 (0.03)	0.17 (0.04)	0.20	2.68	589.38	0.008*	0.80	0.05	0.432
**subcortical**	*R hippocampus*	0.26 (0.03)	0.24 (0.04)	**0.41**	5.16	551.72	< 0.001*	1.00	0.14	**0.015**
	*L hippocampus*	0.25 (0.03)	0.24 (0.03)	**0.36**	4.33	567.38	< 0.001*	1.00	0.17	**0.004**
	*R accumbens*	0.03 (0.01)	0.03 (0.01)	**0.36**	3.56	582.62	< 0.001*	0.97	–0.03	0.564
	*L accumbens*	0.03 (0.01)	0.02 (0.01)	**0.31**	2.88	572.95	0.004*	0.90	–0.03	0.583
	*R ventral diencephalon*	0.25 (0.02)	0.24 (0.03)	**0.30**	3.41	583.70	0.001*	0.96	0.09	0.107
	*R amygdala*	0.11 (0.01)	0.10 (0.02)	0.29	3.96	533.19	< 0.001*	1.00	0.09	0.124
	*L amygdala*	0.09 (0.02)	0.09 (0.02)	0.28	2.93	571.85	0.004*	0.91	0.09	0.104
	*R putamen*	0.29 (0.03)	0.28 (0.04)	0.27	3.18	551.64	0.002*	0.97	–0.02	0.733
	*L ventral diencephalon*	0.25 (0.02)	0.24 (0.03)	0.23	2.59	586.93	0.010*	0.79	0.10	0.085
**temporal**	*R temporal plane of the superior temporal G*	0.10 (0.02)	0.09 (0.02)	**0.32**	3.87	591.56	< 0.001*	0.97	–0.02	0.696
	*R temporal pole*	0.40 (0.05)	0.38 (0.07)	**0.31**	4.01	562.92	< 0.001*	1.00	–0.03	0.559
	*L temporal pole*	0.37 (0.06)	0.35 (0.07)	**0.30**	3.54	581.13	< 0.001*	0.97	–0.07	0.217
	*R lateral aspect of the superior temporal G*	0.32 (0.04)	0.31 (0.05)	**0.30**	3.47	565.91	0.001*	0.98	0.03	0.658
	*R superior temporal S*	0.57 (0.08)	0.55 (0.09)	0.24	2.98	578.14	0.003*	0.91	–0.01	0.910
	*L anterior transverse temporal G*	0.06 (0.01)	0.06 (0.01)	0.20	2.52	586.87	0.012*	0.77	–0.07	0.229
	*R anterior transverse temporal G*	0.05 (0.01)	0.05 (0.01)	0.20	2.69	592.81	0.007*	0.77	–0.04	0.512
	*L inferior temporal G*	0.43 (0.07)	0.41 (0.07)	0.17	2.87	591.19	0.004*	0.85	0.07	0.235

*Significant after FDR correction. Values in **bold** are significant at *p* < 0.05

^†^negative *d* values indicate volumes that are larger in converters than non-converters (FDR corrected), positive values indicate volumes that are smaller in converters than non-converters, and those in **bold** are medium (rather than small) effect sizes

*d* Cohen’s *d*; *df* degrees of freedom for the *t*-test; 1−*β* power; *r* Spearman’s *r, G* gyrus, *S* sulcus, *L* left, *R* right

**Table 4:** Differences between converters and non-converters’ brain saliencies and their correlations with time to conversion. Only structures that survived FDR correction are listed

**lobe**	**structure**	**non-converters** ***μ(σ)*** ^†^	**converters** ***μ(σ)*** ^‡^	***d***	***t***	***df***	***p***	**1−*β***	***r***	***p***
**frontal**	*R orbital sulci*	10.33 (3.65)	**9.62 (3.51)**	**0.18**	**2.42**	**590.69**	0.016	**0.66**	–0.21	<0.001*
	*R middle frontal G*	7.72 (3.48)	7.26 (4.34)	0.27	1.44	571.82	0.150	0.37	–0.18	0.001*
	*R medial orbital S*	9.34 (3.00)	8.82 (2.98)	0.20	2.13	592.52	**0.033**	0.57	–0.18	0.002*
	*R subcentral G and sulci*	8.40 (2.92)	8.16 (3.36)	0.11	0.95	585.16	0.343	0.17	–0.17	0.003*
**limbic**	*R anterior part of the cingulate G and S*	6.50 (1.92)	6.25 (2.09)	0.16	1.54	591.02	0.125	0.36	–0.19	0.001*
**occipital**	*R cuneus*	4.09 (1.56)	4.16 (1.58)	–0.04	–0.58	592.93	0.564	0.09	–0.18	0.001*
	*L middle occipital S and lunatus S*	2.92 (1.32)	2.92 (1.32)	–0.02	0.01	592.71	0.992	0.05	–0.17	0.003*
**parietal**	*R precuneus*	2.96 (0.96)	3.03 (1.08)	–0.04	–0.84	587.40	0.404	0.14	–0.18	0.002*
**subcortical**	*R amygdala*	8.47 (2.86)	7.86 (2.64)	0.21	2.71	586.87	**0.007**	0.74	–0.18	0.001*
**temporal**	*R inferior temporal G*	8.48 (2.27)	8.34 (2.32)	0.06	0.74	593.00	0.460	0.12	–0.21	<0.001*
	*L temporal plane of the superior temporal G*	3.97 (1.41)	3.90 (1.86)	0.12	0.47	558.51	0.636	0.09	–0.18	0.002*
**white matter**	*R cerebral white matter*	5.25 (1.19)	5.10 (1.25)	0.11	1.52	592.72	0.129	0.34	–0.18	0.002*

*Significant after FDR correction. Values in bold are significant at *p* < 0.05

^†^Values in this column are divided by 10^-04^

^‡^Values in this column are divided by 10^-05^

*d* Cohen’s *d*; *df* degrees of freedom for the *t*-test; 1−*β* power; *r* Spearman’s *r, G* gyrus, *S* sulcus, *L* left, *R* right

#### Saliency is correlated with time to conversion

**Fig. 3:**
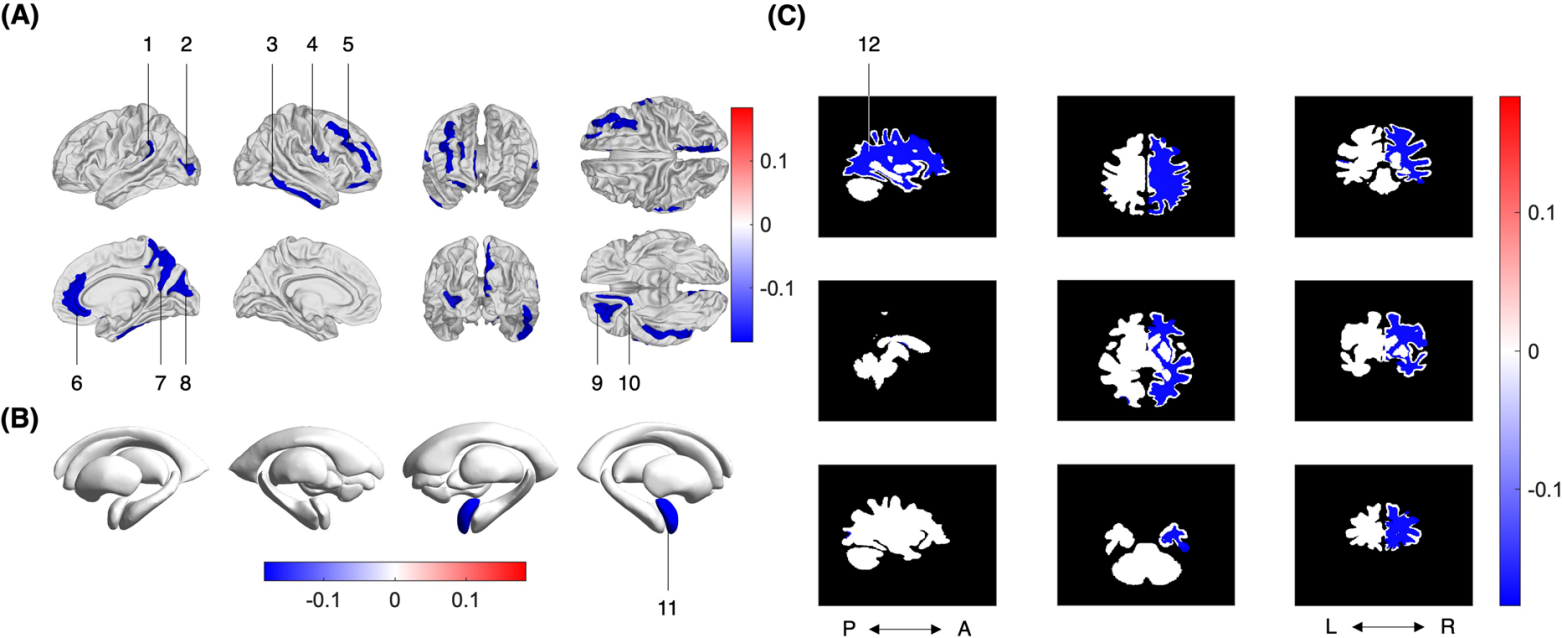
**A** Cortical, **B** subcortical, B & C cerebrospinal fluid, and (C) white matter structures where brain age saliency is significantly correlated with time to conversion (after FDR correction), pictured in neurological convention. Spearman correlations range from *r* = -0.17 (*p* = 0.003; right subcentral gyrus and sulci) to *r* = -0.21 (*p* < 0.001; right orbital sulci), indicating that higher saliencies of these structures are associated with longer time to conversion. 1 = left middle occipital sulcus and lunatus sulcus; 2 = left temporal plane of the superior temporal gyrus; 3 = right inferior temporal gyrus; 4 = right subcentral gyrus and sulci; 5 = right middle frontal gyrus; 6 = right anterior part of the cingulate gyrus and sulcus; 7 = right precuneus; 8 = right cuneus; 9 = right orbital sulci; 10 = right medial orbital sulcus; 11 = right amygdala; 12 = right cerebral white matter

Time to conversion was associated with the saliencies of 12 brain structures (Table 4), including 4 frontal, 2 temporal, 2 occipital, 1 parietal, and 1 limbic (Fig. 3(A)), with the right amygdala (Fig. 3(B)), and with the right cerebral white matter (Fig. 3(C)). In other words, when estimating BA, subjects in whom these structures had DNN features more indicative of age were closer to converting to MCI or AD than subjects in whom these structures were less indicative of age. On average, these 12 regions amount to 2.5% of the TICV. The strongest correlations were observed in the right orbital sulci (*r* = -0.21, *p* < 0.001) and in the right inferior temporal gyrus (*r* = -0.21, *p* < 0.001). Of 12 structures whose saliencies correlated with time to conversion, 10 were in the right hemisphere.

#### Short-term converters are identified more accurately than long-term converters

**Fig. 4:**
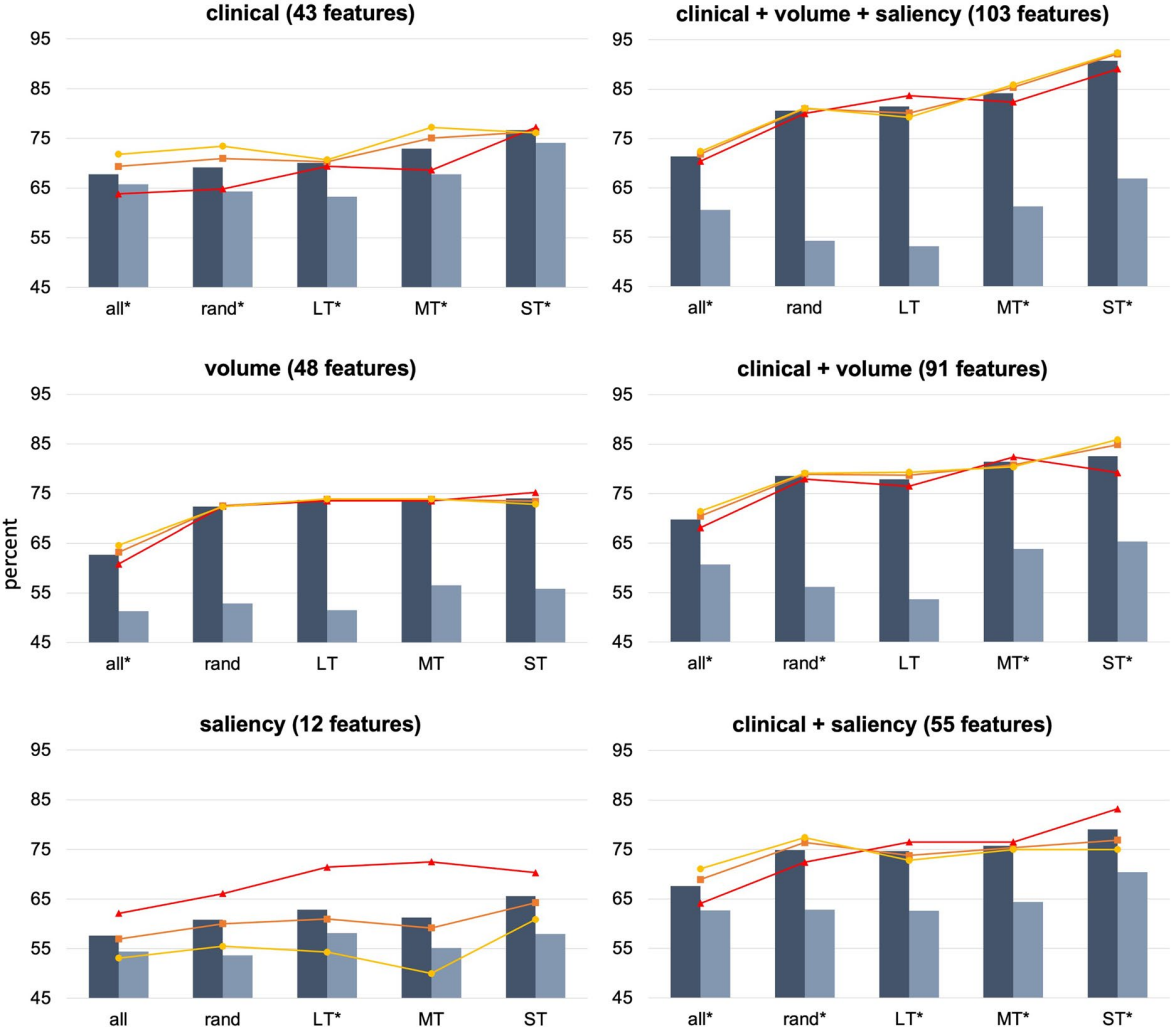
Classification results from linear discriminant analyses (LDAs), including accuracies (dark grey bars), cross-validated accuracies (light grey bars), precisions (orange lines), sensitivities (red lines), and specificities (yellow lines). all = all subjects (i.e., 301 converters and 294 non-converters); rand = 100 converters and 98 non-converters randomly selected and averaged across 3 batches to match the sample sizes of short-term (ST; N = 101), mid-term (MT; N = 102), and long-term (LT; N = 98) converters’, and a sample of matched non-converters (N = 92). Asterisks indicate significant LDAs. All values are reported in full in Supplementary Table E

The results of the best performing LDA (*features:* clinical + volume + saliency, *group:* ST converters) are detailed here. For all other LDAs, the reader can consult Fig. 4 and Supplementary Table E for classification results, and Supplementary Materials 2 for Wilks’ $\lambda$, *χ*^2^, associated *p*-values, and structure matrices. LDAs indicated by asterisks in Fig. 4 and Supplementary Table E were statistically significant, i.e., the discriminant function did better than chance at separating converters from non-converters. The LDA for ST converters that had clinical measures, brain volumes, and BA saliencies as predictors was statistically significant (Wilks’ $\lambda$ = 0.391, *χ*^2^_(72)_ = 138.861, *p* < 0.001). The canonical correlation was 0.78, indicating that the discriminant function explained more than 78% of variance in the relationship between clinical/brain features and group membership. Classification demonstrated that 90.75% of subjects were correctly classified (sensitivity = 89.10%, specificity = 92.40%). However, after leave-one-out cross-validation, only 66.90% of cases were correctly classified (sensitivity = 65.30%, specificity = 68.50%), suggesting overfitting of the original discriminant function. Predictors in this LDA with the highest importance for classification were self-reported memory decline (*r* = 0.328), trails A (*r* = 0.233) and B (*r* = 0.305) time, delayed story recall (*r* = -0.270), verbal fluency (vegetables: *r* = -0.287, animals: *r* = -0.218), left and right hippocampal volumes (left: *r* = -0.203, right: *r* = -0.242), left nucleus accumbens volume (*r* = -0.221), picture naming (*r* = -0.215), left and right ventral diencephalon volumes (left: *r* = -0.200, right: *r* = -0.214), and the volume of the right middle frontal gyrus (*r* = -0.201).

Classification accuracy improved with the combination of neuroimaging and clinical measures (Fig. 4 and Supplementary Table E). Looking only at classification of ST converters from non-converters, classification accuracy using clinical features alone was 76.65% (sensitivity = 77.20%, cross-validated accuracy = 74.10%), volume alone was 74.00% (sensitivity = 75.20%, cross-validated accuracy = 55.85%) and saliency alone was 65.60% (sensitivity = 70.30%, cross-validated accuracy = 57.95%). With clinical and volume features combined, accuracy increased to 82.55% (sensitivity = 79.20%, cross-validated accuracy = 65.35%), and to 79.10% (sensitivity = 83.20%, cross-validated accuracy = 70.45%) with clinical and saliency features combined. Combining clinical, volume, and saliency features resulted in 90.75% classification accuracy (sensitivity = 89.10%, cross-validated accuracy = 66.90%).

Classification accuracy improved as time to conversion decreased, especially for ST converters (Supplementary Table F). Across all LDAs (clinical, volume, clinical + volume, etc.), ST converters were classified from non-converters with an average accuracy of 78.11%, which was 4.67% higher than LT converters’ average accuracy of 73.44%, and 3.25% higher than MT converters’ 74.86%. The difference in average classification accuracy between MT and LT converters (1.42%, on average) was minimal. Sensitivity similarly improved as proximity to conversion approached; on average, 75.17% of LT converters, 75.98% of MT converters, and 79.03% of ST converters were correctly identified.

## Discussion

Thanks to breakthroughs in anti-amyloid therapeutics, clinicians can now improve the prognosis of patients with AD [44]. However, treatment initiation during the pre-clinical or early clinical phase of CI may be most effective [45]. When evaluating CI risk in older adults who are *currently* CN, the development of tools that do not require the presence of CI symptoms is a major goal of current AD research. The results of our study demonstrate that neuroimaging markers enhance the identification of those who will convert to CI, when used in conjunction with clinical indicators of CI risk. Importantly, we find that proximity to conversion influences the accuracy of such identification, as subjects within 2.42 to 5.17 years of conversion are identified more accurately than those 5.18 to 15 years away.

### Progression of neurodegeneration and prediction of CI conversion

The highest accuracies were achieved for ST converters whose conversion was less than ~ 2.5 years away, compared to those whose conversion was ~ 2.5 to 15 years away (MT and LT converters). Classification accuracy decreased as time to conversion increased, suggesting fewer neurodegenerative changes the further one is from CI conversion. Identification of LT converters from macroscopic MRI is inexact because these converters are neurologically similar to non-converters of the same age and sex. Compared to LT converters, ST converters have experienced 12.58 to 15 more years of neurodegeneration than LT converters have yet to witness, making the latter easier to identify. It is possible that conversion may be avoidable for LT converters, assuming that neurodegeneration can be conceptualized as a cumulative process with a point of criticality beyond which CI cannot be avoided. We found, and others have shown, that approximately 11% of patients with a diagnosis of MCI revert to CN status [7, 46]. This suggests that the AD continuum is, at least in its early stages, bidirectional. The environment [47], cognitive training/intervention [48], social dynamics [49], and other factors [50] likely play a role in determining the outcome of processes at work during the crucial interval when at-risk older adults are “on the precipice” of CI. If the outcome of such processes is favorable, it may be unrealistic to expect MRI measures to predict conversion accurately more than ~ 2.5 to ~ 5 years in advance when, in fact, one’s brain trajectory toward CI is reversible.

Predictive models that have quantified *future* CI conversion in CN adults using macroscopic MRI measures (i.e., regional brain volumes) have also included other biomarkers such as those obtained from invasive lumbar punctures [e.g., 9, 16, 51]. For example, Albert et al. [9] found that, in a sample of 46 converters, CSF amyloid-beta and phosphorylated-tau, alongside hippocampal and entorhinal volume, cognitive test scores, and ApoE genotype, can predict conversion up to 5 years beforehand with 80% sensitivity. Aside from this study and a few others [52, 53], previous prediction models did not attempt to distinguish CN converters from CN non-converters [e.g., 18, 19, 20, for review, see 21], possibly because of low model accuracy during cross-validation (which we also encountered, see Sect. "Limitations").

### Brain centers of memory age more in converters than in non-converters

MRI-derived features of disease progression in older adults with CI have been studied extensively [54, 55], but are less clear in older adults who are currently CN. In addition to clinical variables, brain volumes and saliencies improved the accuracy of distinguishing converters from non-converters using LDA [see also 52]. Compared to non-converters, converters exhibit greater-than-typical atrophy across 13% of the brain, including subcortical, temporal, frontal, and parietal grey matter. Converters with higher saliency of frontal and temporal regions in the right hemisphere were closer to conversion than those with lower saliency. This suggests that pronounced conversion-related neurodegeneration of certain regional structures only manifests itself *close* to the onset of symptoms, perhaps more so in the right (subdominant) hemisphere than in the left. Brain structures whose volumes and saliencies were related to conversion include many regions previously associated with CI [3, 11, 14, 56–61], and are detailed in Supplementary Discussion 1.

### Clinical signs of future CI

According to our findings, CN older adults are more likely to convert to CI if they (A) exhibit symptoms of depression, (B) have had subjective memory decline over the previous year, (C) have close family or friends who also report memory decline, and (D) have a hearing impairment. The latter is unsurprising, given that ~ 8% of all-cause dementia risk is explained by hearing loss [1], and that the incidence of dementias is 61% higher among individuals with moderate or severe hearing loss [62]. Hearing loss, which is considered a modifiable risk factor for AD [1], can exacerbate CI risk by increasing the cognitive load of auditory processing [63], by depriving the brain of sensory input and thus reducing brain structure and integrity [64], and/or by reducing social engagement in cognitively stimulating activities [65].

Older adults are also more likely to convert to CI [5] if they self-report a decline in their memory over the previous year [66], and if their close families or friends report a decline in the older adult’s memory [67]. Across all LDAs, self-reported memory decline was among the strongest predictors of conversion. In contrast, ‘objective’ or clinician-endorsed memory impairment was unrelated to future conversion [68]. Prior to CI conversion, older adults may have good insight into their rate of memory decline, noticing deficits in themselves that a clinician might miss at an annual or bi-annual visit [69]. Symptoms of depression, a modifiable risk factor for AD, were more frequent among converters than non-converters. Whether this controls, or is mediated by, converters’ higher incidence of memory complaints is of clinical interest [70] but is beyond the scope of the current study.

### Limitations

We and others have observed that White Hispanic (and Black/African American) older adults are more likely to convert to CI [71]. However, only 24% of our participants were non-White or White Hispanic. Adequate representation of racial and ethnic minorities and accessibility to research opportunities are serious issues given that a staggering 92% of MCI cases go undiagnosed [72]. Racial/ethnic minorities, including Hispanic individuals of any ancestry, are under-represented in AD research [73, 74], including in the NACC dataset. NACC participants are also skewed towards the higher education strata, and thus may have higher cognitive reserve than the general population [75]. Future studies should examine the generalizability of our findings to diverse populations.

Our sample of 301 converters was larger than most previous studies investigating CI conversion [e.g., 22, 23, 24]. However, this sample was still too small to inspire certainty that the LD functions had not been overfit. This may have a role in causing our moderate/poor cross-validation accuracies. In future studies, larger samples can be achieved, and results validated, by joint analysis of large longitudinal cohorts (e.g., Alzheimer’s disease neuroimaging initiative ‘ADNI’, Washington Heights/Inwood Columbia aging project ‘WHICAP’). Classification of converters and non-converters could be improved by (A) using more sophisticated ML methods (e.g., neural networks), (B) deriving other measures from MRI and/or more clinical data, and (C) accounting for other CI risk factors such as genetic markers, blood assays, and CSF biomarkers. Time to conversion was not measured precisely, as its recorded value depends on the timing of participants’ examinations. ST, MT, and LT cut-offs were determined based on balancing our data’s sample sizes rather than using a priori principles. Thus, results may vary across other samples. A small percentage (11.62%) of converters reverted to CN after their CI diagnosis; future studies with larger samples should investigate how defining these subjects as ‘converters’ impacts classification accuracy. Finally, we performed binary classification (converters vs. non-converters), rather than quantifying risk for conversion or predicting time to conversion. Future studies should explore these variables because future CI conversion is a dynamic outcome that is oversimplified by binary variable coding [e.g., 52]. For instance, a model that could track risk for future CI could do so over time, quantifying risk for future conversion increasing/decreasing with age, or as an older adult adopts preventative strategies.

### Conclusion

Our work addresses the challenge of predicting future CI conversion among CN older adults, which would allow clinicians to identify those who will benefit most from lifestyle changes and therapeutic interventions that circumvent, prevent, or reverse cognitive deterioration towards AD. First, neuroanatomic brain features apparent on MRI can enhance the accuracy of cognitive, demographic, and health measures in identifying converters to CI. Alongside clinical symptoms such as hearing impairment, self-reported memory decline, and depressive symptoms, smaller brain volumes and higher regional age saliencies are more apparent in converters compared to non-converters. Second, aging of the right amygdala, white matter, and of the frontal and temporal cortices accelerates as older adults approach conversion. Third, converter identification is most accurate in older adults who are within ~ 2.5 years of conversion. Neuroimaging biomarkers appear to be less helpful prior to this time, potentially highlighting cumulative uncertainty in neurodegeneration trajectories, and supporting the hypothesis that conversion to CI may be preventable prior to this crucial period.

## Competing Interests

The authors have no competing interests to declare that are relevant to the content of this article.

## Supplementary Information

Below is the link to the electronic supplementary material.

Supplementary file1 (DOCX 107 KB)

Supplementary file2 (XLSX 85 KB)

## Data Availability

Qualified researchers may obtain access to all de-identified imaging data and demographic and cognitive used for this study here: https://naccdata.org/.
