# Supplementary material for: Neuroanatomical and clinical factors predicting future cognitive impairment
Source: GeroScience. 2024 Aug 17;47(1):915–34. doi: 10.1007/s11357-024-01310-0 (PMC11872856; doi:10.1007/s11357-024-01310-0)
Supplement: Supplementary file 1 — Supplementary file1 (DOCX 107 KB) [file 11357_2024_1310_MOESM1_ESM.docx]

| Supplementary Table A. Clinical variables | | | | | |
| --- | --- | --- | --- | --- | --- |
| variable | **label** | **form** | **UDS question** | **scale** | **literature** |
| *demographics* | | | | | |
| *age* | AGE | Subject demographics (A1) | Scan year and month minus birth year and month | years: 18 to 110 (cont.) | Livingstone et al. (2020); Xu et al. (2020) |
| *age gap* | AG | N/A | Brain age minus chronological age | years: -18 to 30 (cont.) | Gaser et al. (2013) |
| *sex* | SEX | Subject demographics (A1) | Subject's sex | 1 = male, 2 = female | Altmann et al. (2014) |
| *years of education* | EDUC | Subject demographics (A1) | Years of education | years: 0 to 36 (cont.) | Livingstone et al. (2020); Xu et al. (2020) |
| *race/ethnicity* | RACE_ETH | Subject demographics (A1) | Combination of race and Hispanic variables | 1 = White, 2 = Black or African American, 3 = American Indian or Alaska Native, 4 = Native Hawaiian or Other Pacific Islander, 5 = Asian, 6 = Hispanic White, 7 = Hispanic African American, 8 = Hispanic Native American/Alaskan, 9 = Hispanic Asian, 10 = Hispanic other or unknown, 11 = other | Lin et al. (2021); Mehta & Yeo (2017) |
| *marital status* | MARISTAT | Subject demographics (A1) | Marital status | 1 = Married , 2 = Widowed , 3 = Divorced , 4 = Separated , 5 = Never married (or marriage was annulled) , 6 = Living as married/domestic partner, 9 = Other or unknown | Livingstone et al. (2020); Xu et al. (2020) |
| *independence of living* | INDEPEND | Subject demographics (A1) | Level of independence | 1 = Able to live independently , 2 = Requires some assistance with complex activities , 3 = Requires some assistance with basic activities , 4 = Completely dependent , 9 = Unknown | Pang et al. (2023) |
| *handedness* | HANDED | Subject demographics (A1) | Is the subject left- or right-handed? | 1 = Left-handed , 2 = Right-handed , 3 = Ambidextrous , 9 = Unknown | Doody et al. (1999) |
| *health* | | | | | |
| *smoked in last 30 days* | TOBAC30 | Subject health history (A5) | Smoked cigarettes in last 30 days | 0 = No, 1 = Yes  9 = Unknown | Livingstone et al. (2020); Pang et al. (2023) |
| *history of heart attack* | CVHATT | Subject health history (A5) | Heart attack/cardiac arrest | 0 = Absent, 1 = Recent/Active, 2 = Remote/Inactive, 9 = Unknown | Livingstone et al. (2020) |
| *history of stroke* | CBSTROKE | Subject health history (A5) | Stroke | 0 = Absent, 1 = Recent/Active, 2 = Remote/Inactive, 9 = Unknown | Livingstone et al. (2020) |
| *history of TBI* | NACCTBI | Subject health history (A5) | History of traumatic brain injury (TBI) | 0 = No, 1 = Yes, 9 = Unknown | Livingstone et al. (2020) |
| *history of diabetes* | DIABETES | Subject health history (A5) | Diabetes | 0 = Absent, 1 = Recent/Active, 2 = Remote/Inactive, 9 = Unknown | Livingstone et al. (2020) |
| *history of hypertension* | HYPERTEN | Subject health history (A5) | Hypertension | 0 = Absent, 1 = Recent/Active, 2 = Remote/Inactive, 9 = Unknown | Livingstone et al. (2020) |
| *history of alcohol abuse* | ALCOHOL | Subject health history (A5) | Alcohol abuse - clinically significant impairment occurring over a 12-month period manifested in one of the following areas: work, driving, legal, or social | 0 = Absent, 1 = Recent/Active, 2 = Remote/Inactive, 9 = Unknown | Livingstone et al. (2020) |
| *hearing impairment* | HEARING | Physical (B1) | Without a hearing aid(s), is the subject's hearing functionally normal? | 0 = No, 1 = Yes, 9 = Unknown | Livingstone et al. (2020) |
| *body mass index* | NACCBMI | Physical (B1) | Body mass index (BMI) | Index: 10.0 to 100.0 (cont.) | Livingstone et al. (2020) |
| *geriatric depression scale* | NACCGDS | Geriatric depression scale (B6) | Total GDS Score | Score: 0 to 15 (cont.) | Livingstone et al. (2020) |
| *apoE genotype* | NACCNE4S | RDD-Gen | Number of APOE e4 alleles | 0 = No e4 allele, 1 = 1 copy of e4 allele, 2 = 2 copies of e4 allele, 9 = Missing/unknown/not assessed | Pang et al. (2023) |
| *interview-based measures of neurocognitive function* | | | | | |
| *memory impairment (clinician assessed)* | MEMORY | CDR® Plus NACC FTLD (B4) | Memory scale (from the CDR) | 0.0 = No impairment,  0.5 = Questionable impairment, 1.0 = Mild impairment,  2.0 = Moderate impairment, 3.0 = Severe impairment | Pang et al. (2023) |
| *judgment (clinician assessed)* | JUDGMENT | CDR® Plus NACC FTLD (B4) | Judgment and problem-solving (from the CDR) | 0.0 = No impairment,  0.5 = Questionable impairment, 1.0 = Mild impairment,  2.0 = Moderate impairment, 3.0 = Severe impairment | Pang et al. (2023) |
| *community engagement (clinician assessed)* | COMMUN | CDR® Plus NACC FTLD (B4) | Community affairs (from the CDR) | 0.0 = No impairment,  0.5 = Questionable impairment, 1.0 = Mild impairment,  2.0 = Moderate impairment, 3.0 = Severe impairment | Pang et al. (2023) |
| *global CDR* | CDRGLOB | CDR® Plus NACC FTLD (B4) | Global CDR score | 0.0 = No impairment,  0.5 = Questionable impairment, 1.0 = Mild impairment,  2.0 = Moderate impairment, 3.0 = Severe impairment | Pang et al. (2023) |
| *memory impairment (self-reported)* | DECSUB | Clinician judgment of symptoms (B9) | Does the subject report a decline in memory (relative to previously attained abilities)? | 0 = No, 1 = Yes, 8 = Could not be assessed/subject too impaired | Pang et al. (2023) |
| *memory impairment (informant-reported)* | DECIN | Clinician judgment of symptoms (B9) | Does the co-participant report a decline in subject's memory (relative to previously attained abilities)? | 0 = No, 1 = Yes, 8 = There is no co-participant, 9 = Unknown | Pang et al. (2023) |
| *visuospatial function (clinician assessed)* | COGVIS | Clinician judgment of symptoms (B9) | Indicate whether the subject currently is meaningfully impaired, relative to previously attained abilities, in visuospatial function | 0 = No, 1 = Yes, 9 = Unknown | Pang et al. (2023) |
| *first symptom of cognitive decline* | NACCCOGF | Clinician judgment of symptoms (B9) | Indicate the predominant symptom that was first recognized as a decline in the subject's cognition | 0 = No impairment in cognition, 1 = Memory, 2 = Orientation, 3 = Executive function - judgment, planning, problem-solving, 4 = Language, 5 = Visuospatial function, 6 = Attention/concentration, 7 = Fluctuating cognition, 8 = Other | Pang et al. (2023) |
| *mode of onset of cognitive decline* | COGMODE | Clinician judgment of symptoms (B9) | Mode of onset of cognitive symptoms | 0 = No impairment in cognition, 1 = Gradual, 2 = Subacute, 3 = Abrupt, 4 = Other (specify) | Pang et al. (2023) |
| *first symptom of behavioural impairment* | NACCBEHF | Clinician judgment of symptoms (B9) | Indicate the predominant symptom that was first recognized as a decline in the subject's behavior | 0 = No behavioral symptoms, 1 = Apathy/withdrawal, 2 = Depressed mood, 3 = Psychosis, 4 = Disinhibition, 5 = Irritability, 6 = Agitation, 7 = Personality change, 8 = REM sleep behavior disorder, 9 = Anxiety, 10 = Other (specify) | Pang et al. (2023) |
| *mode of onset of behavioural impairment* | BEMODE | Clinician judgment of symptoms (B9) | Mode of onset of behavioral symptoms | 0 = No behavioral symptoms, 1 = Gradual, 2 = Subacute, 3 = Abrupt, 4 = Other (specify) | Pang et al. (2023) |
| *first symptom of motor impairment* | NACCMOTF | Clinician judgment of symptoms (B9) | Indicate the predominant symptom that was first recognized as a decline in the subject's motor function | 0 = No motor symptoms, 1 = Gait disorder, 2 = Falls, 3 = Tremor, 4 = Slowness | Pang et al. (2023) |
| *mode of onset of motor impairment* | MOMODE | Clinician judgment of symptoms (B9) | Mode of onset of motor symptoms | 0 = No motor symptoms, 1 = Gradual, 2 = Subacute, 3 = Abrupt, 4 = Other | Pang et al. (2023) |
| *cognitive status* | COGSTAT | Neuropsychological battery summary scores (C1, C2, C2T) | Per the clinician, based on the UDS  neuropsychological examination, the  subject's cognitive status is deemed: | 0 = Clinician unable to render opinion, 1 = Better than normal for age, 2 = Normal for age, 3 = One or two test scores abnormal, 4 = Three or more scores are abnormal or lower than expected | Pang et al. (2023) |
| *neurocognitive test scores* | | | | | |
| *story recall (immediate)* | CRAFTVRS_LOGIMEM_COMBINED | Neuropsychological battery scores (C1, C2, C2T) | Craft story 21 recall immediate – Total story units recalled (CRAFTVRS, UDS v3.0 and v3.2) and Logical memory IA immediate – Total number of story units recalled (LOGIMEM, UDS v1.2 and v2.0) combined | Percentage of total possible correct units recalled (correctly recalled units/total possible units*100) (cont.)  CRAFTVRS: 0 to 44  LOGIMEM: 0 to 25 | Pang et al. (2023) |
| *story recall (delayed)* | CRAFTDVR_MEMUNITS_COMBINED | Neuropsychological battery scores (C1, C2, C2T) | Craft story 21 recall delayed – Total story units recalled (CRAFTDVR, UDS v3.0 and v3.2) and Logical memory IIA delayed – Total number of story units recalled (MEMUNITS, UDS v1.2 and v2.0) combined | Percentage of total possible correct units recalled (correctly recalled units/total possible units*100) (cont.)  CRAFTDVR: 0 to 44  MEMUNITS: 0 to 25 | completeness |
| *story recall (delay time)* | CRAFTDTI_MEMTIME_COMBINED | Neuropsychological battery scores (C1, C2, C2T) | Craft story 21 recall delayed – Delay time (CRAFTDTI, UDS v3.0 and v3.2) and Logical memory IIA delayed – time elapsed since logical memory IA immediate (MEMTIME, UDS v1.2 and v2.0) combined | Minutes: 0 to 85 (cont.) | Pang et al. (2023) |
| *digits forward* | DIGIFLEN_DIGFORSL_COMBINED | Neuropsychological battery scores (C1, C2, C2T) | Digit span forwards - Length (DIGIFLEN, UDS v1.2 and v2.0) and Number span test - Longest span forwards (DIGFORSL, UDS v3.0 and v3.2) combined | Percentage of total possible correct unit span (longest span/total possible span*100) (cont.)  DIGIFLEN: 0 to 8  DIGFORSL: 3 to 9 | completeness |
| *digits backward* | DIGIBLEN_DIGBACLS_COMBINED | Neuropsychological battery scores (C1, C2, C2T) | Digit span backwards - Length (DIGIBLEN, UDS v1.2 and v2.0) and Number span test - Longest span backwards (DIGBACLS, UDS v3.0 and v3.2) combined | Percentage of total possible correct unit span (longest span/total possible span*100) (cont.)  DIGIBLEN: 0 to 8  DIGBACLS: 2 to 8 | Pang et al. (2023) |
| *verbal fluency (animals)* | ANIMALS | Neuropsychological battery scores (C1, C2, C2T) | Animals - total number of animals named  in 60 seconds | Count: 0 to 77 (cont.) | Pang et al. (2023) |
| *verbal fluency (vegetables)* | VEG | Neuropsychological battery scores (C1, C2, C2T) | Vegetables - total number of vegetables  named in 60 seconds | Count: 0 to 77 (cont.) | Pang et al. (2023) |
| *trails A* | TRAILA | Neuropsychological battery scores (C1, C2, C2T) | Trail Making Test Part A - total number  of seconds to complete | Time (seconds): 0 to 150 (cont.) | Pang et al. (2023) |
| *trails B* | TRAILB | Neuropsychological battery scores (C1, C2, C2T) | Trail Making Test Part B - total  number of seconds to complete | Time (seconds): 0 to 300 (cont.) | completeness |
| *picture naming* | BOSTON_MINTTOTS_COMBINED | Neuropsychological battery scores (C1, C2, C2T) | Multilingual naming test - total score (MINTTOTS, UDS v3.0 and v3.2) and Boston naming test - total score (BOSTON, UDS v1.2 and 2.0) combined | Percentage of total possible correct units named (correctly named units/total possible units*100) (cont.)  MINTTOTS: 0 to 32  BOSTON: 0 to 30 | Pang et al. (2023) |
| Note: variables are categorical unless indicated in the scale column as “cont.” | | | | | |

| **Supplementary Table B.** Brain structure labels, volume types, and names | | | |
| --- | --- | --- | --- |
|  | **Freesurfer label** | **volume type** | **name** |
| 1 | rh_G_and_S_frontomargin_volume | frontal | right frontomarginal gyrus and sulcus |
| 2 | rh_G_and_S_subcentral_volume | frontal | right subcentral gyrus and sulci |
| 3 | rh_G_and_S_transv_frontopol_volume | frontal | right transverse frontopolar gyri and sulci |
| 4 | rh_G_front_inf_Opercular_volume | frontal | right opercular part of the inferior frontal gyrus |
| 5 | rh_G_front_inf_Orbital_volume | frontal | right orbital part of the inferior frontal gyrus |
| 6 | rh_G_front_inf_Triangul_volume | frontal | right triangular part of the inferior frontal gyrus |
| 7 | rh_G_front_middle_volume | frontal | right middle frontal gyrus |
| 8 | rh_G_front_sup_volume | frontal | right superior frontal gyrus |
| 9 | rh_G_orbital_volume | frontal | right orbital gyri |
| 10 | rh_G_precentral_volume | frontal | right precentral gyrus |
| 11 | rh_G_rectus_volume | frontal | right straight gyrus |
| 12 | rh_Lat_Fis_ant_Horizont_volume | frontal | right horizontal ramus of the anterior segment of the lateral sulcus |
| 13 | rh_Lat_Fis_ant_Vertical_volume | frontal | right vertical ramus of the anterior segment of the lateral sulcus |
| 14 | rh_Lat_Fis_post_volume | frontal | right posterior ramus of the lateral sulcus |
| 15 | rh_S_front_inf_volume | frontal | right inferior frontal sulcus |
| 16 | rh_S_front_middle_volume | frontal | right middle frontal sulcus |
| 17 | rh_S_front_sup_volume | frontal | right superior frontal sulcus |
| 18 | rh_S_orbital_lateral_volume | frontal | right lateral orbital sulcus |
| 19 | rh_S_orbital_med_olfact_volume | frontal | right medial orbital sulcus |
| 20 | rh_S_orbital_H_Shaped_volume | frontal | right orbital sulci |
| 21 | rh_S_precentral_inf_part_volume | frontal | right inferior part of the precentral sulcus |
| 22 | rh_S_precentral_sup_part_volume | frontal | right superior part of the precentral sulcus |
| 23 | rh_S_suborbital_volume | frontal | right suborbital sulcus |
| 24 | lh_G_and_S_frontomargin_volume | frontal | left frontomarginal gyrus and sulcus |
| 25 | lh_G_and_S_subcentral_volume | frontal | left subcentral gyrus and sulci |
| 26 | lh_G_and_S_transv_frontopol_volume | frontal | left transverse frontopolar gyri and sulci |
| 27 | lh_G_front_inf_Opercular_volume | frontal | left opercular part of the inferior frontal gyrus |
| 28 | lh_G_front_inf_Orbital_volume | frontal | left orbital part of the inferior frontal gyrus |
| 29 | lh_G_front_inf_Triangul_volume | frontal | left triangular part of the inferior frontal gyrus |
| 30 | lh_G_front_middle_volume | frontal | left middle frontal gyrus |
| 31 | lh_G_front_sup_volume | frontal | left superior frontal gyrus |
| 32 | lh_G_orbital_volume | frontal | left orbital gyri |
| 33 | lh_G_precentral_volume | frontal | left precentral gyrus |
| 34 | lh_G_rectus_volume | frontal | left straight gyrus |
| 35 | lh_Lat_Fis_ant_Horizont_volume | frontal | left horizontal ramus of the anterior segment of the lateral sulcus |
| 36 | lh_Lat_Fis_ant_Vertical_volume | frontal | left vertical ramus of the anterior segment of the lateral sulcus |
| 37 | lh_Lat_Fis_post_volume | frontal | left posterior ramus of the lateral sulcus |
| 38 | lh_S_front_inf_volume | frontal | left inferior frontal sulcus |
| 39 | lh_S_front_middle_volume | frontal | left middle frontal sulcus |
| 40 | lh_S_front_sup_volume | frontal | left superior frontal sulcus |
| 41 | lh_S_orbital_lateral_volume | frontal | left lateral orbital sulcus |
| 42 | lh_S_orbital_med_olfact_volume | frontal | left medial orbital sulcus |
| 43 | lh_S_orbital_H_Shaped_volume | frontal | left orbital sulci |
| 44 | lh_S_precentral_inf_part_volume | frontal | left inferior part of the precentral sulcus |
| 45 | lh_S_precentral_sup_part_volume | frontal | left superior part of the precentral sulcus |
| 46 | lh_S_suborbital_volume | frontal | left suborbital sulcus |
| 47 | rh_S_central_volume | frontal/parietal | right central sulcus |
| 48 | lh_S_central_volume | frontal/parietal | left central sulcus |
| 49 | rh_G_and_S_cingul_Ant_volume | limbic | right anterior part of the cingulate gyrus and sulcus |
| 50 | rh_G_and_S_cingul_Mid_Ant_volume | limbic | right middle-anterior part of the cingulate gyrus and sulcus |
| 51 | rh_G_and_S_cingul_Mid_Post_volume | limbic | right middle posterior part of the cingulate gyrus and sulcus |
| 52 | rh_G_cingul_Post_dorsal_volume | limbic | right posterior-dorsal part of the cingulate gyrus |
| 53 | rh_G_cingul_Post_ventral_volume | limbic | right posterior-ventral part of the cingulate gyrus |
| 54 | rh_G_Ins_lg_and_S_cent_ins_volume | limbic | right long insular gyrus and central sulcus of the insula |
| 55 | rh_G_insular_short_volume | limbic | right short insular gyri |
| 56 | rh_G_oc_temp_med_Parahip_volume | limbic | right parahippocampal gyrus |
| 57 | rh_G_subcallosal_volume | limbic | right subcallosal gyrus |
| 58 | rh_S_circular_insula_ant_volume | limbic | right anterior part of the circular sulcus of the insula |
| 59 | rh_S_circular_insula_inf_volume | limbic | right inferior part of the circular sulcus of the insula |
| 60 | rh_S_circular_insula_sup_volume | limbic | right superior part of the circular sulcus of the insula |
| 61 | rh_S_pericallosal_volume | limbic | right pericallosal sulcus |
| 62 | lh_G_and_S_cingul_Ant_volume | limbic | left anterior part of the cingulate gyrus and sulcus |
| 63 | lh_G_and_S_cingul_Mid_Ant_volume | limbic | left middle-anterior part of the cingulate gyrus and sulcus |
| 64 | lh_G_and_S_cingul_Mid_Post_volume | limbic | left middle posterior part of the cingulate gyrus and sulcus |
| 65 | lh_G_cingul_Post_dorsal_volume | limbic | left posterior-dorsal part of the cingulate gyrus |
| 66 | lh_G_cingul_Post_ventral_volume | limbic | left posterior-ventral part of the cingulate gyrus |
| 67 | lh_G_Ins_lg_and_S_cent_ins_volume | limbic | left long insular gyrus and central sulcus of the insula |
| 68 | lh_G_insular_short_volume | limbic | left short insular gyri |
| 69 | lh_G_oc_temp_med_Parahip_volume | limbic | left parahippocampal gyrus |
| 70 | lh_G_subcallosal_volume | limbic | left subcallosal gyrus |
| 71 | lh_S_circular_insula_ant_volume | limbic | left anterior part of the circular sulcus of the insula |
| 72 | lh_S_circular_insula_inf_volume | limbic | left inferior part of the circular sulcus of the insula |
| 73 | lh_S_circular_insula_sup_volume | limbic | left superior part of the circular sulcus of the insula |
| 74 | lh_S_pericallosal_volume | limbic | left pericallosal sulcus |
| 75 | rh_G_and_S_occipital_inf_volume | occipital | right inferior occipital gyrus and sulcus |
| 76 | rh_G_cuneus_volume | occipital | right cuneus |
| 77 | rh_G_occipital_middle_volume | occipital | right middle occipital gyrus |
| 78 | rh_G_occipital_sup_volume | occipital | right superior occipital gyrus |
| 79 | rh_G_oc_temp_lat_fusifor_volume | occipital | right lateral occipito-temporal gyrus |
| 80 | rh_G_oc_temp_med_Lingual_volume | occipital | right lingual gyrus |
| 81 | rh_Pole_occipital_volume | occipital | right occipital pole |
| 82 | rh_S_calcarine_volume | occipital | right calcarine sulcus |
| 83 | rh_S_collat_transv_post_volume | occipital | right posterior transverse collateral sulcus |
| 84 | rh_S_oc_middle_and_Lunatus_volume | occipital | right middle occipital sulcus and lunatus sulcus |
| 85 | rh_S_oc_sup_and_transversal_volume | occipital | right superior occipital sulcus and transverse occipital sulcus |
| 86 | rh_S_occipital_ant_volume | occipital | right anterior occipital sulcus |
| 87 | rh_S_oc_temp_lat_volume | occipital | right lateral occipito-temporal sulcus |
| 88 | rh_S_oc_temp_med_and_Lingual_volume | occipital | right medial occipito-temporal sulcus and lingual sulcus |
| 89 | lh_G_and_S_occipital_inf_volume | occipital | left inferior occipital gyrus and sulcus |
| 90 | lh_G_cuneus_volume | occipital | left cuneus |
| 91 | lh_G_occipital_middle_volume | occipital | left middle occipital gyrus |
| 92 | lh_G_occipital_sup_volume | occipital | left superior occipital gyrus |
| 93 | lh_G_oc_temp_lat_fusifor_volume | occipital | left lateral occipito-temporal gyrus |
| 94 | lh_G_oc_temp_med_Lingual_volume | occipital | left lingual gyrus |
| 95 | lh_Pole_occipital_volume | occipital | left occipital pole |
| 96 | lh_S_calcarine_volume | occipital | left calcarine sulcus |
| 97 | lh_S_collat_transv_post_volume | occipital | left posterior transverse collateral sulcus |
| 98 | lh_S_oc_middle_and_Lunatus_volume | occipital | left middle occipital sulcus and lunatus sulcus |
| 99 | lh_S_oc_sup_and_transversal_volume | occipital | left superior occipital sulcus and transverse occipital sulcus |
| 100 | lh_S_occipital_ant_volume | occipital | left anterior occipital sulcus |
| 101 | lh_S_oc_temp_lat_volume | occipital | left lateral occipito-temporal sulcus |
| 102 | lh_S_oc_temp_med_and_Lingual_volume | occipital | left medial occipito-temporal sulcus and lingual sulcus |
| 103 | rh_G_and_S_paracentral_volume | parietal | right paracentral lobule and sulcus |
| 104 | rh_G_pariet_inf_Angular_volume | parietal | right angular gyrus |
| 105 | rh_G_pariet_inf_Supramar_volume | parietal | right supramarginal gyrus |
| 106 | rh_G_parietal_sup_volume | parietal | right superior partiel gyrus |
| 107 | rh_G_postcentral_volume | parietal | right postcentral gyrus |
| 108 | rh_G_precuneus_volume | parietal | right precuneus |
| 109 | rh_S_cingul_Marginalis_volume | parietal | right marginal branch of the cingulate sulcus |
| 110 | rh_S_interm_prim_Jensen_volume | parietal | right sulcus intermediate primus |
| 111 | rh_S_intrapariet_and_P_trans_volume | parietal | right intraparietal sulcus and transverse parietal sulci |
| 112 | rh_S_postcentral_volume | parietal | right postcentral sulcus |
| 113 | rh_S_subparietal_volume | parietal | right subparietal sulcus |
| 114 | lh_G_and_S_paracentral_volume | parietal | left paracentral lobule and sulcus |
| 115 | lh_G_pariet_inf_Angular_volume | parietal | left angular gyrus |
| 116 | lh_G_pariet_inf_Supramar_volume | parietal | left supramarginal gyrus |
| 117 | lh_G_parietal_sup_volume | parietal | left superior partiel gyrus |
| 118 | lh_G_postcentral_volume | parietal | left postcentral gyrus |
| 119 | lh_G_precuneus_volume | parietal | left precuneus |
| 120 | lh_S_cingul_Marginalis_volume | parietal | left marginal branch of the cingulate sulcus |
| 121 | lh_S_interm_prim_Jensen_volume | parietal | left sulcus intermediate primus |
| 122 | lh_S_intrapariet_and_P_trans_volume | parietal | left intraparietal sulcus and transverse parietal sulci |
| 123 | lh_S_postcentral_volume | parietal | left postcentral sulcus |
| 124 | lh_S_subparietal_volume | parietal | left subparietal sulcus |
| 125 | rh_S_parieto_occipital_volume | parietal/occipital | right parieto-occipital sulcus |
| 126 | lh_S_parieto_occipital_volume | parietal/occipital | left parieto-occipital sulcus |
| 127 | rh_G_temp_sup_G_T_transv_volume | temporal | right anterior transverse temporal gyrus |
| 128 | rh_G_temp_sup_Lateral_volume | temporal | right lateral aspect of the superior temporal gyrus |
| 129 | rh_G_temp_sup_Plan_polar_volume | temporal | right planum polare of the superior temporal gyrus |
| 130 | rh_G_temp_sup_Plan_tempo_volume | temporal | right temporal plane of the superior temporal gyrus |
| 131 | rh_G_temporal_inf_volume | temporal | right inferior temporal gyrus |
| 132 | rh_G_temporal_middle_volume | temporal | right middle temporal gyrus |
| 133 | rh_Pole_temporal_volume | temporal | right temporal pole |
| 134 | rh_S_collat_transv_ant_volume | temporal | right anterior transverse collateral sulcus |
| 135 | rh_S_temporal_inf_volume | temporal | right inferior temporal sulcus |
| 136 | rh_S_temporal_sup_volume | temporal | right superior temporal sulcus |
| 137 | rh_S_temporal_transverse_volume | temporal | right transverse temporal sulcus |
| 138 | lh_G_temp_sup_G_T_transv_volume | temporal | left anterior transverse temporal gyrus |
| 139 | lh_G_temp_sup_Lateral_volume | temporal | left lateral aspect of the superior temporal gyrus |
| 140 | lh_G_temp_sup_Plan_polar_volume | temporal | left planum polare of the superior temporal gyrus |
| 141 | lh_G_temp_sup_Plan_tempo_volume | temporal | left temporal plane of the superior temporal gyrus |
| 142 | lh_G_temporal_inf_volume | temporal | left inferior temporal gyrus |
| 143 | lh_G_temporal_middle_volume | temporal | left middle temporal gyrus |
| 144 | lh_Pole_temporal_volume | temporal | left temporal pole |
| 145 | lh_S_collat_transv_ant_volume | temporal | left anterior transverse collateral sulcus |
| 146 | lh_S_temporal_inf_volume | temporal | left inferior temporal sulcus |
| 147 | lh_S_temporal_sup_volume | temporal | left superior temporal sulcus |
| 148 | lh_S_temporal_transverse_volume | temporal | left transverse temporal sulcus |
| 149 | Left_Cerebellum_Cortex_volume | subcortical | left cerebellar cortex |
| 150 | Left_Thalamus_volume | subcortical | left thalamus |
| 151 | Left_Caudate_volume | subcortical | left caudate |
| 152 | Left_Putamen_volume | subcortical | left putamen |
| 153 | Left_Pallidum_volume | subcortical | left pallidum |
| 154 | Brain_Stem_volume | subcortical | brainstem |
| 155 | Left_Hippocampus_volume | subcortical | left hippocampus |
| 156 | Left_Amygdala_volume | subcortical | left amygdala |
| 157 | Left_Accumbens_area_volume | subcortical | left accumbens |
| 158 | Left_VentralDC_volume | subcortical | left ventral diencephalon |
| 159 | Right_Cerebellum_Cortex_volume | subcortical | right cerebellum cortex |
| 160 | Right_Thalamus_volume | subcortical | right thalamus |
| 161 | Right_Caudate_volume | subcortical | right caudate |
| 162 | Right_Putamen_volume | subcortical | right putamen |
| 163 | Right_Pallidum_volume | subcortical | right pallidum |
| 164 | Right_Hippocampus_volume | subcortical | right hippocampus |
| 165 | Right_Amygdala_volume | subcortical | right amygdala |
| 166 | Right_Accumbens_area_volume | subcortical | right accumbens |
| 167 | Right_VentralDC_volume | subcortical | right ventral diencephalon |
| 168 | Left_Lateral_Ventricle_volume | CSF system | left lateral ventricle |
| 169 | Left_Inf_Lat_Vent_volume | CSF system | left inferior lateral ventricle |
| 170 | 3rd_Ventricle_volume | CSF system | 3rd ventricle |
| 171 | 4th_Ventricle_volume | CSF system | 4th ventricle |
| 172 | CSF_volume | CSF system | cerebrospinal fluid |
| 173 | Left_choroid_plexus_volume | CSF system | left choroid plexus |
| 174 | Right_Lateral_Ventricle_volume | CSF system | right lateral ventricle |
| 175 | Right_Inf_Lat_Vent_volume | CSF system | right inferior lateral ventricle |
| 176 | Right_choroid_plexus_volume | CSF system | right choroid plexus |
| 177 | Left_Cerebellum_White_Matter_volume | white matter | left cerebellar white matter |
| 178 | Right_Cerebellum_White_Matter_volume | white matter | right cerebellum white matter |
| 179 | CC_Posterior_volume | white matter | posterior corpus callosum |
| 180 | CC_Mid_Posterior_volume | white matter | middle-posterior corpus callosum |
| 181 | CC_Central_volume | white matter | central corpus callosum |
| 182 | CC_Mid_Anterior_volume | white matter | middle-anterior corpus callosum |
| 183 | CC_Anterior_volume | white matter | anterior corpus callosum |
| 184 | Left_Cerebral_White_Matter_volume | white matter | left cerebral white matter |
| 185 | Right_Cerebral_White_Matter_volume | white matter | right cerebral white matter |
| 1 to 148 | cortical_GM_volume | total | cortical grey matter |
| 149 to 167 | subcortical_GM_volume | total | subcortical grey matter |
| 177 to 185 | WM_volume | total | white matter |
| 168 to 176 | CSF_system_volume | total | cerebrospinal fluid system |

| **Supplementary Table C.** Comparison of clinical AD-risk factors between converters and non-converters | | | | | | | | | | |
| --- | --- | --- | --- | --- | --- | --- | --- | --- | --- | --- |
|  |  | **non-converters** | | | **converters** | | | χ² | | |
|  |  | *OC* | *EC* | *%* | *OC* | *EC* | *%* | χ² | *df* | *p* |
| ***demographics*** | | | | | | | | | | |
| **sex** |  |  |  |  |  |  |  | 0.041 | 1 | 0.840 |
|  | *male* | 109 | 110.2 | 37.1↓ | 114 | 112.8 | 37.9↑ |  |  |  |
|  | *female* | 185 | 183.8 | 62.9↑ | 187 | 188.2 | 62.1↓ |  |  |  |
| **handedness** |  |  |  |  |  |  |  | 3.076 | 3 | 0.380 |
|  | *left-handed* | 21 | 21.7 | 7.1↓ | 23 | 22.3 | 7.6↑ |  |  |  |
|  | *right-handed* | 267 | 266.8 | 90.8↑ | 273 | 273.2 | 90.7↓ |  |  |  |
| ***** | *ambidextrous* | 6 | 4.4 | 2.0 | 3 | 4.5 | 1.0 |  |  |  |
| ***** | *unknown* | 0 | 1.0 | 0.0 | 2 | 1.0 | 0.7 |  |  |  |
| **race/ethnicity** |  |  |  |  |  |  |  | **17.320** | **7** | **.015** |
|  | *White non-Hispanic* | 228 | 223.8 | 77.6↑ | 224 | 228.7 | 74.4↓ | 0.221 | 1 | 0.6381 |
|  | *Black or African American* | 42 | 36.6 | 14.3↑ | 32 | 37.4 | 10.6↓ | 1.635 | 1 | 0.201 |
| ***** | *American Indian or Alaska Native* | 3 | 2.5 | 1.0 | 2 | 2.5 | 0.7 | 0.2 | 1 | 0.6547 |
|  | *Asian* | 3 | 6.4 | 1.0↓ | 10 | 6.6 | 3.3↑ | 3.558 | 1 | 0.0593 |
|  | ***White Hispanic*** | **14** | **22.7** | **4.8↓** | **32** | **23.3** | **10.6↑** | **6.583** | **1** | **0.0103** |
| ***** | *Black or African American Hispanic* | 1 | 0.5 | 0.3 | 0 | 0.5 | 0.0 | 1 | 1 | 0.3173 |
| ***** | *Hispanic other/unknown* | 0 | 0.5 | 0.0 | 1 | 0.5 | 0.3 | 1 | 1 | 0.3173 |
| ***** | *other/unknown* | 3 | 1.5 | 1.0 | 0 | 1.5 | 0.0 | 2 | 1 | 0.1573 |
| **marital status** |  |  |  |  |  |  |  | 9.496 | 6 | 0.148 |
|  | *married* | 174 | 165.5 | 59.2↑ | 161 | 169.5 | 53.5↓ |  |  |  |
|  | *widowed* | 52 | 62.3 | 17.7↓ | 74 | 63.7 | 24.6↑ |  |  |  |
|  | *divorced* | 41 | 37.6 | 13.9↑ | 35 | 38.4 | 11.6↓ |  |  |  |
| ***** | *separated* | 0 | 2.0 | 0.0 | 4 | 2.0 | 1.3 |  |  |  |
|  | *never married* | 20 | 20.3 | 6.8↓ | 21 | 20.7 | 7.0↑ |  |  |  |
| ***** | *domestic partnership* | 6 | 4.9 | 2.0 | 4 | 5.1 | 1.3 |  |  |  |
| ***** | *other/unknown* | 1 | 1.5 | 0.3 | 2 | 1.5 | 0.7 |  |  |  |
| **independence of living** |  |  |  |  |  |  |  | 1.027 | 2 | 0.598 |
|  | *lives independently* | 289 | 289.6 | 98.3↓ | 297 | 296.4 | 98.7↑ |  |  |  |
| ***** | *requires assistance with complex activities* | 4 | 4.0 | 1.4 | 4 | 4.0 | 1.3 |  |  |  |
| ***** | *requires assistance with basic activities* | 1 | 0.5 | 0.3 | 0 | 0.5 | 0.0 |  |  |  |
| ***health*** | | | | | | | | | | |
| **apoE genotype** |  |  |  |  |  |  |  | **7.165** | **2** | **0.028** |
|  | *zero e4s* | 210 | 201.1 | 71.4↑ | 197 | 205.9 | 65.4↓ | 0.779 | 1 | 0.378 |
|  | *one e4* | 81 | 85.0 | 27.6↓ | 91 | 87.0 | 30.2↑ | 0.372 | 1 | 0.542 |
|  | ***two e4s*** | **3** | **7.9** | **1.0↓** | **13** | **8.1** | **4.3↑** | **6.003** | **1** | **0.014** |
| **hearing impairment** |  |  |  |  |  |  |  | **4.747** | **1** | **0.029** |
|  | ***yes*** | **73** | **86.0** | **25.4↓** | **101** | **88.0** | **33.7↑** | **3.886** | **1** | **0.049** |
|  | *no* | 214 | 202.0 | 74.6↑ | 197 | 209.0 | 66.3↓ | 1.402 | 1 | 0.236 |
| **smoked in last 30 days** |  |  |  |  |  |  |  | 1.699 | 1 | 0.192 |
|  | *no* | 285 | 282.1 | 97.6↑ | 287 | 289.9 | 95.7↓ |  |  |  |
|  | *yes* | 7 | 9.9 | 2.4↓ | 13 | 10.1 | 4.3↑ |  |  |  |
| **abused alcohol in lifetime** |  |  |  |  |  |  |  | 1.724 | 2 | 0.422 |
|  | *absent* | 285 | 283.1 | 97.3↑ | 289 | 290.9 | 96.0↓ |  |  |  |
| *** | *recent/active* | 2 | 1.5 | 0.7 | 1 | 1.5 | 0.3 |  |  |  |
|  | *remote/inactive* | 6 | 8.4 | 2.0↓ | 11 | 8.6 | 3.7↑ |  |  |  |
| **history of cardiac arrest** |  |  |  |  |  |  |  | 1.773 | 2 | 0.412 |
|  | *absent* | 283 | 281.2 | 96.6↑ | 287 | 288.8 | 95.3↓ |  |  |  |
| *** | *recent/active* | 1 | 2.5 | 0.3 | 4 | 2.5 | 1.3 |  |  |  |
|  | *remote/inactive* | 9 | 9.4 | 3.1↓ | 10 | 9.6 | 3.3↑ |  |  |  |
| **history of stroke** |  |  |  |  |  |  |  | 1.016 | 2 | .060 |
|  | *absent* | 290 | 290.5 | 98.6↓ | 295 | 294.5 | 99.0↑ |  |  |  |
| *** | *recent/active* | 1 | 0.5 | 0.3 | 0 | 0.5 | 0.0 |  |  |  |
| *** | *remote/inactive* | 3 | 3.0 | 1.0 | 3 | 3.0 | 1.0 |  |  |  |
| **history of hypertension** |  |  |  |  |  |  |  | 1.241 | 2 | 0.538 |
|  | *absent* | 153 | 151.7 | 52.0↑ | 154 | 155.3 | 51.2↓ |  |  |  |
|  | *recent/active* | 134 | 132.9 | 45.6↑ | 135 | 136.1 | 44.9↓ |  |  |  |
|  | *remote/inactive* | 7 | 9.4 | 2.4↓ | 12 | 9.6 | 4.0↑ |  |  |  |
| **history of diabetes** |  |  |  |  |  |  |  | 0.095 | 2 | 0.954 |
|  | *absent* | 262 | 261.0 | 90.0↑ | 268 | 269.0 | 89.3↓ |  |  |  |
|  | *recent/active* | 26 | 27.1 | 8.9↓ | 29 | 27.9 | 9.7↑ |  |  |  |
| *** | *remote/inactive* | 3 | 3.0 | 1.0 | 3 | 3.0 | 1.0 |  |  |  |
| **history of traumatic brain injury** |  |  |  |  |  |  |  | 0.214 | 1 | 0.644 |
|  | *no* | 258 | 259.8 | 88.1↑ | 266 | 264.2 | 89.3↓ |  |  |  |
|  | *yes* | 35 | 33.2 | 11.9↓ | 32 | 33.8 | 10.7↑ |  |  |  |
| ***interview-based measures of neurocognitive functioning*** | | | | | | | | | | |
| **cognitive status** |  |  |  |  |  |  |  | **22.023** | **4** | **<0.001*** |
| ***** | *unable to render opinion* | 6 | 4.4 | 2.0 | 3 | 4.6 | 1.0 | 1.138 | 1 | 0.286 |
|  | ***better than normal for age*** | **54** | **36.6** | **18.4↑** | **20** | **37.4** | **6.6↓** | **16.367** | **1** | **<0.001*** |
|  | *normal for age* | 179 | 187.3 | 60.9↑ | 200 | 191.7 | 66.4↓ | 0.727 | 1 | 0.394 |
|  | *1-2 abnormal test scores* | 51 | 59.8 | 17.3↓ | 70 | 61.2 | 23.3↑ | 2.560 | 1 | 0.110 |
|  | *> 3 abnormal test scores* | 4 | 5.9 | 1.4↓ | 8 | 6.1 | 2.7↑ | 1.204 | 1 | 0.273 |
| **CDR (global)** |  |  |  |  |  |  |  | 2.460 | 2 | 0.292 |
|  | *no impairment* | 251 | 245.1 | 85.4↑ | 245 | 250.9 | 81.4↓ |  |  |  |
|  | *questionable impairment* | 43 | 48.4 | 14.6↓ | 55 | 49.6 | 18.3↑ |  |  |  |
| ***** | *mild impairment* | 0 | 0.5 | 0.0 | 1 | 0.5 | 0.3 |  |  |  |
| **memory (clinician assessed)** |  |  |  |  |  |  |  | 5.888 | 2 | 0.053 |
|  | *no impairment* | 252 | 245.6 | 85.7↑ | 245 | 251.4 | 81.4↓ |  |  |  |
|  | *questionable impairment* | 42 | 46.0 | 14.3↓ | 51 | 47.0 | 16.9↑ |  |  |  |
| ***** | *mild impairment* | 0 | 2.5 | 0.0 | 5 | 2.5 | 1.7 |  |  |  |
| **memory impairment (self-reported)** |  |  |  |  |  |  |  | **21.329** | **1** | **<0.001*** |
|  | ***no*** | **214** | **187.1** | **73.5**↑ | **166** | **192.9** | **55.3**↓ | **7.619** | **1** | **0.006*** |
|  | ***yes*** | **77** | **103.9** | **26.5**↓ | **134** | **107.1** | **44.7**↑ | **13.721** | **1** | **<0.001*** |
| **memory impairment (informant-reported)** |  |  |  |  |  |  |  | **14.111** | **1** | **<0.001*** |
|  | *no* | 246 | 228.4 | 87.2↑ | 218 | 235.6 | 74.9↓ | 2.671 | 1 | 0.102 |
|  | ***yes*** | **36** | **53.6** | **12.8**↓ | **73** | **55.4** | **25.1**↑ | **11.370** | **1** | **<0.001*** |
| **judgment (clinician assessed)** |  |  |  |  |  |  |  | 3.676 | 2 | 0.159 |
|  | *no impairment* | 273 | 268.8 | 92.9↑ | 271 | 275.2 | 90.0↓ |  |  |  |
|  | *questionable impairment* | 21 | 23.7 | 7.1↓ | 27 | 24.3 | 9.0↑ |  |  |  |
| ***** | *mild impairment* | 0 | 1.5 | 0.0 | 3 | 1.5 | 1.0 |  |  |  |
| **community engagement (clinician assessed)** |  |  |  |  |  |  |  | 1.267 | 1 | 0.260 |
|  | *no impairment* | 290 | 288.1 | 98.6↑ | 293 | 294.9 | 97.3↓ |  |  |  |
|  | *questionable impairment* | 4 | 5.9 | 1.4↓ | 8 | 6.1 | 2.7↑ |  |  |  |
| **visuospatial function (clinician assessed)** |  |  |  |  |  |  |  | 0.960 | 1 | 0.327 |
|  | *not meaningfully impaired* | 293 | 292.0 | 99.7↑ | 298 | 299.0 | 99.0↓ |  |  |  |
| ***** | *meaningfully impaired* | 1 | 2.0 | 0.3 | 3 | 2.0 | 1.0 |  |  |  |
|  |  |  |  |  |  |  |  |  |  |  |
| **first symptom of cognitive impairment** |  |  |  |  |  |  |  | 3.929 | 4 | 0.416 |
|  | *no impairment* | 277 | 271.3 | 94.2↑ | 272 | 277.7 | 90.4↓ |  |  |  |
|  | *memory* | 14 | 18.8 | 4.8↓ | 24 | 19.2 | 8.0↑ |  |  |  |
| ***** | *executive function* | 1 | 1.5 | 0.3 | 2 | 1.5 | 0.7 |  |  |  |
| ***** | *language* | 0 | 0.5 | 0.0 | 1 | 0.5 | 0.3 |  |  |  |
| ***** | *attention and concentration* | 2 | 2.0 | 0.7 | 2 | 2.0 | 0.7 |  |  |  |
| **mode of onset of cognitive impairment** |  |  |  |  |  |  |  | 2.799 | 2 | 0.247 |
|  | *no impairment* | 277 | 271.3 | 94.2↑ | 272 | 277.3 | 90.7↓ |  |  |  |
|  | *gradual* | 16 | 21.3 | 5.4↓ | 27 | 21.7 | 9.0↑ |  |  |  |
|  | *abrupt* | 1 | 1.0 | 0.3 | 1 | 1.0 | 0.3 |  |  |  |
| **first symptom of behavioural impairment** |  |  |  |  |  |  |  | 6.254 | 5 | 0.282 |
|  | *no impairment* | 282 | 276.2 | 96.9↑ | 276 | 281.8 | 92.9↓ |  |  |  |
| ***** | *apathy and withdrawal* | 1 | 2.5 | 0.3 | 4 | 2.5 | 1.3 |  |  |  |
|  | *depressed mood* | 5 | 7.9 | 1.7↓ | 11 | 8.1 | 3.7↑ |  |  |  |
| ***** | *irritability* | 2 | 2.5 | 0.7 | 3 | 2.5 | 1.0 |  |  |  |
| ***** | *personality change* | 1 | 1.0 | 0.3 | 1 | 1.0 | 0.3 |  |  |  |
| ***** | *other* | 0 | 1.0 | 0.0 | 2 | 1.0 | 0.7 |  |  |  |
| **mode of onset of behavioural impairment** |  |  |  |  |  |  |  | 8.380 | 4 | 0.079 |
|  | *no impairment* | 282 | 276.1 | 96.2↑ | 275 | 280.9 | 92.3↓ |  |  |  |
|  | *gradual* | 7 | 13.9 | 2.4↓ | 21 | 14.1 | 7.0↑ |  |  |  |
| ***** | *subacute* | 2 | 1.5 | 0.7 | 1 | 1.5 | 0.3 |  |  |  |
| ***** | *abrupt* | 1 | 1.0 | 0.3 | 1 | 1.0 | 0.3 |  |  |  |
| ***** | *other* | 1 | 0.5 | 0.3 | 0 | 0.5 | 0.0 |  |  |  |
| **first symptom of motor impairment** |  |  |  |  |  |  |  | 4.001 | 3 | 0.261 |
|  | *no impairment* | 290 | 290.0 | 98.6 | 296 | 296.0 | 98.7 |  |  |  |
| ***** | *gait disorder* | 2 | 2.0 | 0.7 | 2 | 2.0 | 0.7 |  |  |  |
| ***** | *tremor* | 2 | 1.0 | 0.7 | 0 | 1.0 | 0.7 |  |  |  |
| ***** | *slowness* | 0 | 1.0 | 0.0 | 2 | 1.0 | 0.7 |  |  |  |
| **mode of onset of motor impairment** |  |  |  |  |  |  |  | 0.162 | 1 | 0.687 |
|  | *no impairment* | 290 | 290.5 | 98.6↓ | 296 | 295.5 | 99.0↑ |  |  |  |
| ***** | *gradual* | 4 | 3.5 | 1.4 | 3 | 3.5 | 1.0 |  |  |  |
| Note: OC = observed counts, EC = expected counts. * Some expected counts in this row are less than 5, and χ² calculations are only reliable when all the expected values are 5 or higher. Green↑ indicates a percentage that is larger than expected, and red ↓ a percentage that is smaller than expected, but only those in **bold** are significantly higher/lower than expected. **Bold** indicates significance at *p* < .05, asterisks * indicate significance after FDR correction. Row-wise comparisons were done post-hoc when the omnibus χ² was statistically significant, to determine the source of the association. | | | | | | | | | | |

| Supplementary Table D. Comparison of neuropsychological test scores between non-converters and converters | | | | | | |
| --- | --- | --- | --- | --- | --- | --- |
|  | **non-converters**  **μ (σ)** | **converters**  **μ (σ)** | ***t*** | ***df*** | ***p*** | ***Δ*** |
| *story immediate recall* | 55.92 (15.90) | 48.96 (16.59) ↓ | 5.196 | 587 | **< 0.001*** | 0.419 |
| *story delayed recall* | 50.91 (16.49) | 42.38 (17.70) ↓ | 6.054 | 588 | **< 0.001*** | 0.482 |
| *story delay time* | 20.47 (6.71) | 20.57 (7.01) | -0.186 | 587.211 | 0.853 | -0.015 |
| *digits forward* | 78.67 (14.59) | 77.59 (15.79) | 0.863 | 590 | 0.388 | 0.068 |
| *digits backward* | 63.27 (15.26) | 57.84 (15.52) ↓ | 4.285 | 590 | **< 0.001*** | 0.349 |
| *verbal fluency animals* | 20.95 (5.40) | 19.11 (5.13) ↓ | 4.261 | 593 | **< 0.001*** | 0.359 |
| *verbal fluency vegetables* | 14.78 (4.28) | 13.32 (4.05) ↓ | 4.273 | 591 | **< 0.001*** | 0.361 |
| *trails A* | 32.08 (11.35) | 36.70 (13.55) ↑ | -4.487 | 573.802 | **< 0.001*** | -0.341 |
| *trails B* | 84.39 (42.21) | 107.60 (58.17) ↑ | -5.546 | 540.356 | **< 0.001*** | -0.399 |
| *picture naming* | 93.99 (6.94) | 89.43 (11.79) ↓ | 5.723 | 480.852 | **< 0.001*** | 0.386 |
| Note: **Bold** indicates significance at *p* < 0.05, asterisks * indicate differences were significant after FDR correction. Red indicates on average worse performance in this group, green indicates better performance on average in this group, and the arrows indicate whether scores were higher or lower on average in the converters. Scores for story *immediate recall, story delayed recall, digits forward, digits backward*, and *picture naming* are expressed as the percentage of correct responses of the total possible correct responses. Time is the score value for *story delay time* (minutes), *trails A* (seconds), and *trails B* (seconds). *Verbal fluency animals* and *verbal fluency vegetables* scores are the total count of correct responses. *Δ* is Glass’s delta as an estimate of effect size that does not assume equal variances. Levene’s test for the equality of variances was significant for *story delay time, trails A, trails B,* and *picture naming,* thus equal variances were not assumed for these comparisons. | | | | | | |

| **Supplementary Table E.** Linear discriminant analysis classification accuracies for converters and non-converters. | | | | | | | | | | | |
| --- | --- | --- | --- | --- | --- | --- | --- | --- | --- | --- | --- |
|  |  |  | **original** | | | |  | **cross-validated** | | | |
| **features (*N*)** | **subs** |  | ***acc*** | ***prec*** | ***sens*** | ***spec*** |  | ***acc*** | ***prec*** | ***sens*** | ***spec*** |
| ***clinical (43)*** | *all** |  | 67.80 | 69.35 | 63.80 | 71.80 |  | 65.75 | 66.84 | 62.50 | 69.00 |
|  | *rand** |  | 69.13 | 70.97 | 64.83 | 73.43 |  | 64.32 | 66.09 | 58.97 | 69.67 |
|  | ***ST**** |  | **76.65** | **76.36** | **77.20** | **76.10** |  | **74.10** | **74.00** | **74.30** | **73.90** |
|  | *MT** |  | 72.90 | 75.05 | 68.60 | 77.20 |  | 67.75 | 69.74 | 62.70 | 72.80 |
|  | *LT** |  | 70.05 | 70.31 | 69.40 | 70.70 |  | 63.30 | 64.49 | 59.20 | 67.40 |
| ***volume (48)*** | *all** |  | 62.70 | 63.20 | 60.80 | 64.60 |  | 51.30 | 51.33 | 50.20 | 52.40 |
|  | *rand* |  | 72.35 | 72.51 | 72.40 | 72.30 |  | 52.92 | 52.52 | 54.13 | 51.70 |
|  | ***ST*** |  | **74.00** | 73.44 | **75.20** | 72.80 |  | 55.85 | 55.57 | 58.40 | 53.30 |
|  | ***MT*** |  | 73.70 | **73.80** | 73.50 | **73.90** |  | **56.55** | **56.27** | **58.80** | **54.30** |
|  | ***LT*** |  | 73.70 | **73.80** | 73.50 | **73.90** |  | 51.55 | 51.54 | 52.00 | 51.10 |
| ***saliency (12)*** | *all* |  | 57.60 | 56.97 | 62.10 | 53.10 |  | 54.40 | 54.07 | 58.50 | 50.30 |
|  | *rand* |  | 60.80 | 60.01 | 66.10 | 55.50 |  | 53.63 | 53.72 | 59.43 | 47.83 |
|  | ***ST*** |  | **65.60** | **64.26** | 70.30 | **60.90** |  | 57.95 | **57.73** | 59.40 | **56.50** |
|  | ***MT*** |  | 61.25 | 59.18 | **72.50** | 50.00 |  | 55.15 | 54.25 | 65.70 | 44.60 |
|  | *LT** |  | 62.85 | 60.97 | 71.40 | 54.30 |  | **58.15** | 57.01 | **66.30** | 50.00 |
| ***clinical +***  ***volume (91)*** | *all** |  | 69.75 | 70.42 | 68.10 | 71.40 |  | 60.70 | 61.19 | 58.50 | 62.90 |
|  | *rand** |  | 78.53 | 78.91 | 77.93 | 79.13 |  | 56.18 | 56.60 | 52.97 | 59.40 |
|  | ***ST**** |  | **82.55** | **84.89** | 79.20 | **85.90** |  | **65.35** | **65.65** | 64.40 | **66.30** |
|  | ***MT**** |  | 81.40 | 80.78 | **82.40** | 80.40 |  | 63.85 | 63.62 | **64.70** | 63.00 |
|  | *LT* |  | 77.90 | 78.70 | 76.50 | 79.30 |  | 53.70 | 53.67 | 54.10 | 53.30 |
| ***clinical +***  ***saliency (55)*** | *all** |  | 67.60 | 68.92 | 64.10 | 71.10 |  | 62.70 | 63.40 | 60.10 | 65.30 |
|  | ***rand**** |  | 74.90 | 76.39 | 72.40 | **77.40** |  | 62.82 | 64.01 | 59.90 | 65.73 |
|  | ***ST**** |  | **79.10** | **76.89** | **83.20** | 75.00 |  | **70.45** | **70.11** | **71.30** | **69.60** |
|  | *MT** |  | 75.75 | 75.37 | 76.50 | 75.00 |  | 64.40 | 64.31 | 64.70 | 64.10 |
|  | *LT** |  | 74.65 | 73.77 | 76.50 | 72.80 |  | 62.60 | 62.19 | 64.30 | 60.90 |
| ***clinical +***  ***volume +***  ***saliency (103)*** | *all** |  | 71.40 | 71.84 | 70.40 | 72.40 |  | 60.55 | 60.78 | 59.50 | 61.60 |
|  | *rand* |  | 80.62 | 81.03 | 80.07 | 81.17 |  | 54.28 | 54.10 | 54.53 | 54.03 |
|  | ***ST**** |  | **90.75** | **92.14** | **89.10** | **92.40** |  | **66.90** | **67.46** | **65.30** | **68.50** |
|  | *MT** |  | 84.15 | 85.39 | 82.40 | 85.90 |  | 61.25 | 60.93 | 62.70 | 59.80 |
|  | *LT* |  | 81.50 | 80.17 | 83.70 | 79.30 |  | 53.20 | 53.21 | 53.10 | 53.30 |
| *all* = all subjects (i.e., 301 converters and 294 non-converters); *rand* = 100 converters and 98 non-converters randomly selected in 3 batches to match the sample sizes of short-term (ST; *N* = 101), mid-term (MT; *N* = 102), and long-term (LT; *N* = 98) converters’, and a sample of matched non-converters (*N* = 92). The average classification measures were taken across the 3 batches. | | | | | | | | | | | |

| **Supplementary Table F.** Comparison of linear discriminant analysis classification accuracies, precisions, sensitivities, and specificities across ST, MT, and LT converters. | | | | | | | | | | | |
| --- | --- | --- | --- | --- | --- | --- | --- | --- | --- | --- | --- |
|  |  |  | **original** | | | |  | **cross-validated** | | | |
|  | **features** |  | ***acc*** | ***prec*** | ***sens*** | ***spec*** |  | ***acc*** | ***prec*** | ***sens*** | ***spec*** |
| ***ST – LT*** | *clinical* |  | 6.6 | 6.05 | 7.8 | 5.4 |  | 10.8 | 9.51 | 15.1 | 6.5 |
|  | *volume* |  | 0.3 | -0.36 | 1.7 | -1.1 |  | 4.3 | 4.03 | 6.4 | 2.2 |
|  | *saliency* |  | 2.75 | 3.29 | -1.1 | 6.6 |  | -0.2 | 0.72 | -6.9 | 6.5 |
|  | *clinical + volume* |  | 4.65 | 6.19 | 2.7 | 6.6 |  | 11.65 | 11.98 | 10.3 | 13 |
|  | *clinical + saliency* |  | 4.45 | 3.12 | 6.7 | 2.2 |  | 7.85 | 7.92 | 7 | 8.7 |
|  | *clinical + volume + saliency* |  | 9.25 | 11.97 | 5.4 | 13.1 |  | 13.7 | 14.25 | 12.2 | 15.2 |
|  | ***average*** |  | **4.67** | **5.04** | **3.87** | **5.47** |  | **8.02** | **8.07** | **7.35** | **8.68** |
| ***ST – MT*** | *clinical* |  | 3.75 | 1.31 | 8.6 | -1.1 |  | 6.35 | 4.26 | 11.6 | 1.1 |
|  | *volume* |  | 0.3 | -0.36 | 1.7 | -1.1 |  | -0.7 | -0.7 | -0.4 | -1 |
|  | *saliency* |  | 4.35 | 5.08 | -2.2 | 10.9 |  | 2.8 | 3.48 | -6.3 | 11.9 |
|  | *clinical + volume* |  | 1.15 | 4.11 | -3.2 | 5.5 |  | 1.5 | 2.03 | -0.3 | 3.3 |
|  | *clinical + saliency* |  | 3.35 | 1.52 | 6.7 | 0 |  | 6.05 | 5.8 | 6.6 | 5.5 |
|  | *clinical + volume + saliency* |  | 6.6 | 6.75 | 6.7 | 6.5 |  | 5.65 | 6.53 | 2.6 | 8.7 |
|  | ***average*** |  | **3.25** | **3.07** | **3.05** | **3.45** |  | **3.61** | **3.57** | **2.30** | **4.92** |
| ***MT – LT*** | *clinical* |  | 2.85 | 4.74 | -0.8 | 6.5 |  | 4.45 | 5.25 | 3.5 | 5.4 |
|  | *volume* |  | 0 | 0 | 0 | 0 |  | 5 | 4.73 | 6.8 | 3.2 |
|  | *saliency* |  | -1.6 | -1.79 | 1.1 | -4.3 |  | -3 | -2.76 | -0.6 | -5.4 |
|  | *clinical + volume* |  | 3.5 | 2.08 | 5.9 | 1.1 |  | 10.15 | 9.95 | 10.6 | 9.7 |
|  | *clinical + saliency* |  | 1.1 | 1.6 | 0 | 2.2 |  | 1.8 | 2.12 | 0.4 | 3.2 |
|  | *clinical + volume + saliency* |  | 2.65 | 5.22 | -1.3 | 6.6 |  | 8.05 | 7.72 | 9.6 | 6.5 |
|  | ***average*** |  | **1.42** | **1.98** | **0.82** | **2.02** |  | **4.41** | **4.50** | **5.05** | **3.77** |
| ST – LT = the difference in accuracy/precision/sensitivity/specificity (%) between classifying ST converters from non-converters and LT converters from non-converters (i.e., ST classification accuracy – LT classification accuracy).  ST – MT = the same as above but for ST converters and MT converters  ST – MT = the same as above but for MT converters and LT converters  average = the average accuracy/precision/sensitivity/specificity across all LDAs/feature types | | | | | | | | | | | |

**Supplementary Discussion 1.**

The largest differences in volume were observed in the subcortex, including the bilateral hippocampi (1-6) and accumbens (7-9). Hippocampal atrophy is associated with conversion from CN to MCI (10). Meta-analysis revealed left and right hippocampal volumes are 12.9% and 11.1% lower in persons with MCI, and 24.2% and 23.1% lower respectively in persons with AD (3). Atrophy of the accumbens is a consequence of AD-related degeneration of connected limbic structures (9). Significant differences were also observed in the temporal cortex, including the bilateral temporal poles (11-13) and the right superior temporal gyrus (14, 15). BA saliency of the right inferior temporal gyrus was associated with time to conversion. These three temporal regions all exhibit differences in volume between stable MCI patients and those who will convert to AD (11). Volume of the frontal cortex (bilateral precentral gyri) (16), and saliency of the orbital sulci (13, 17) were implicated in conversion status. Finally, the parietal cortex (right supramarginal gyrus, left marginal branch of the cingulate sulcus, right intraparietal sulcus, and right transverse parietal sulci) (18) exhibited more signs of atrophy in converters compared to non-converters. While medial temporal regions are much touted as the most effective early predictors of AD thanks to neurofibrillary tangle formation starting in the medial temporal lobe (19), amyloid plaques first affect the posterior association cortices (19-21) and metabolic studies show that AD-related dysfunction is more frequent in parietal areas (18, 22). Our results highlight volumetric abnormalities in converters across a range of brain regions, that are not isolated to the temporal or parietal cortices.

References

1. Convit A, De Leon M, Tarshish C, De Santi S, Tsui W, Rusinek H, et al. Specific hippocampal volume reductions in individuals at risk for Alzheimer’s disease. Neurobiology of aging. 1997;18(2):131-8.

2. Jack CR, Shiung M, Weigand S, O’brien P, Gunter J, Boeve B, et al. Brain atrophy rates predict subsequent clinical conversion in normal elderly and amnestic MCI. Neurology. 2005;65(8):1227-31.

3. Shi F, Liu B, Zhou Y, Yu C, Jiang T. Hippocampal volume and asymmetry in mild cognitive impairment and Alzheimer's disease: Meta‐analyses of MRI studies. Wiley Online Library; 2009. p. 1055-64.

4. Schuff N, Woerner N, Boreta L, Kornfield T, Shaw L, Trojanowski J, et al. MRI of hippocampal volume loss in early Alzheimer's disease in relation to ApoE genotype and biomarkers. Brain. 2009;132(4):1067-77.

5. McRae-McKee K, Evans S, Hadjichrysanthou C, Wong M, De Wolf F, Anderson R. Combining hippocampal volume metrics to better understand Alzheimer’s disease progression in at-risk individuals. Scientific reports. 2019;9(1):7499.

6. Uysal G, Ozturk M. Hippocampal atrophy based Alzheimer’s disease diagnosis via machine learning methods. Journal of Neuroscience Methods. 2020;337:108669.

7. Bayassi-Jakowicka M, Lietzau G, Czuba E, Patrone C, Kowiański P. More than addiction: The nucleus accumbens contribution to development of mental disorders and neurodegenerative diseases. International Journal of Molecular Sciences. 2022;23(5):2618.

8. Massett RJ, Maher AS, Imms PE, Amgalan A, Chaudhari NN, Chowdhury NF, et al. Regional neuroanatomic effects on brain age inferred using magnetic resonance imaging and ridge regression. The Journals of Gerontology: Series A. 2023;78(6):872-81.

9. Nie X, Sun Y, Wan S, Zhao H, Liu R, Li X, et al. Subregional structural alterations in hippocampus and nucleus accumbens correlate with the clinical impairment in patients with Alzheimer’s disease clinical spectrum: parallel combining volume and vertex-based approach. Frontiers in neurology. 2017;8:399.

10. Prosser L, Macdougall A, Sudre CH, Manning EN, Malone IB, Walsh P, et al. Predicting Cognitive Decline in Older Adults Using Baseline Metrics of AD Pathologies, Cerebrovascular Disease, and Neurodegeneration. Neurology. 2023;100(8):e834-e45.

11. Risacher SL, Saykin AJ, Wes JD, Shen L, Firpi HA, McDonald BC. Baseline MRI predictors of conversion from MCI to probable AD in the ADNI cohort. Current Alzheimer Research. 2009;6(4):347-61.

12. Arnold SE, Hyman BT, Van Hoesen GW. Neuropathologic changes of the temporal pole in Alzheimer's disease and Pick's disease. Archives of neurology. 1994;51(2):145-50.

13. Nag S, Yu L, Boyle PA, Leurgans SE, Bennett DA, Schneider JA. TDP-43 pathology in anterior temporal pole cortex in aging and Alzheimer’s disease. Acta neuropathologica communications. 2018;6(1):1-11.

14. Convit A, De Asis J, De Leon M, Tarshish C, De Santi S, Rusinek H. Atrophy of the medial occipitotemporal, inferior, and middle temporal gyri in non-demented elderly predict decline to Alzheimer’s disease☆. Neurobiology of aging. 2000;21(1):19-26.

15. Gao Z, Fu HJ, Zhao LB, Sun ZY, Yang YF, Zhu HY. Aberrant DNA methylation associated with Alzheimer's disease in the superior temporal gyrus. Experimental and therapeutic medicine. 2018;15(1):103-8.

16. Humbert IA, McLaren DG, Kosmatka K, Fitzgerald M, Johnson S, Porcaro E, et al. Early deficits in cortical control of swallowing in Alzheimer's disease. Journal of Alzheimer's disease. 2010;19(4):1185-97.

17. Van Hoesen GW, Parvizi J, Chu C-C. Orbitofrontal cortex pathology in Alzheimer's disease. Cerebral Cortex. 2000;10(3):243-51.

18. Jacobs HI, Van Boxtel MP, Jolles J, Verhey FR, Uylings HB. Parietal cortex matters in Alzheimer's disease: an overview of structural, functional and metabolic findings. Neuroscience & Biobehavioral Reviews. 2012;36(1):297-309.

19. Braak H, Braak E. Development of Alzheimer-related neurofibrillary changes in the neocortex inversely recapitulates cortical myelogenesis. Acta neuropathologica. 1996;92:197-201.

20. Braak H, Braak E. Neuropathological stageing of Alzheimer-related changes. Acta Neuropathologica. 1991;82(4):239-59.

21. Thal DR, Rüb U, Orantes M, Braak H. Phases of Aβ-deposition in the human brain and its relevance for the development of AD. Neurology. 2002;58(12):1791-800.

22. Chase TN, Foster NL, Fedio P, Brooks R, Mansi L, di Chiro G. Regional cortical dysfunction in Alzheimer's disease as determined by positron emission tomography. Annals of Neurology: Official Journal of the American Neurological Association and the Child Neurology Society. 1984;15(S1):170-4.
